# Efficacy of acupuncture in animal models of vascular dementia: A systematic review and network meta-analysis

**DOI:** 10.3389/fnagi.2022.952181

**Published:** 2022-08-18

**Authors:** Guangyao Li, Yuling Shi, Lu Zhang, Chuanghui Yang, Ting Wan, Hang Lv, Wenxuan Jian, Jinghu Li, Min Li

**Affiliations:** ^1^Medical College of Acupuncture Moxibustion and Rehabilitation, Guangzhou University of Chinese Medicine, Guangzhou, China; ^2^Science and Technology Innovation Center, Guangzhou University of Chinese Medicine, Guangzhou, China; ^3^The Second Affiliated Hospital, Guangzhou University of Chinese Medicine (Guangdong Hospital of Traditional Chinese Medicine), Guangzhou, China; ^4^The First Affiliated Hospital, Guangzhou University of Chinese Medicine, Guangzhou, China; ^5^Nanfang Hospital, Southern Medical University, Guangzhou, China; ^6^Department of Massage, The Third Affiliated Hospital of Zhejiang Chinese Medical University, Hangzhou, China

**Keywords:** acupuncture, vascular dementia, animal studies, network meta-analysis, morris water maze, acupuncture protocol, acupuncture mechanism

## Abstract

**Background and purpose:**

Acupuncture is widely used in clinical practice for the treatment of vascular diseases. However, the protocol, efficacy, and mechanism of acupuncture in animal models of vascular dementia are still controversial. Based on the above problems, we initiated this comprehensive study.

**Methods:**

To analyze the literatures included in this study, 4 databases were searched and the SYRCLE's Risk of bias tool was employed. To perform the subgroup analysis of different acupuncture methods and the Review Manager 5.3 was applied. Meanwhile, the pairwise and network meta-analysis were conducted using Addis 1.16.8. The outcomes included escape latency, number of crossings, time spent in the target quadrant, and swimming speed.

**Results:**

Forty-two studies with a total of 1,486 animals were included in this meta-analysis. According to the results from subgroup analysis, GV20 + ST36 (Baihui + bilateral Zusanli) combined with 14-day manual acupuncture can obtain best improvement of the rats cognitive function among all acupuncture regimens (MD: −23.41; 95%CI: −26.66, −20.15; I^2^ = 0%; *P* < 0.001). The heterogeneity of other acupuncture treatments was significantly higher than that of GV20 + ST36, because the treatment courses were not uniform. Pair-wise and network comparisons are highly consistent. The major results of the network meta-analysis were as follows, In comparison to the impaired group, the acupuncture group showed significantly reduced escape latency (MD: −25.87; 95%CI: −30.75, −21.12), increased number of original platform crossings (MD: 2.63; 95%CI: 1.94, 3.34) and time spent in the target quadrant (MD: 7.88; 95%CI: 4.25, 11.44). The overall results of the network meta-analysis are as follows: the normal and sham-operated groups performed the best, followed by medicine and acupuncture, while no effect was found in the impaired group treated with non-acupoint and palliative.

**Conclusions:**

Acupuncture significantly improves cognitive function in rats with vascular dementia. Compared to other acupuncture plans, (GV20 + ST36, MA) and 14 -day manual acupuncture can be used to obtain better results. The main mechanism of acupuncture in the treatment of vascular dementia is reduced oxidative stress, neuronal inflammation, and apoptosis, as well as the increased synaptic plasticity and neurotransmitters.

**Systematic review registration:**

https://inplasy.com/inplasy-2021-11-0036/, identifier: INPLASY2021110036.

## Introduction

Vascular dementia (VD) is a syndrome of cognitive dysfunction caused by hypoperfusion disorders in brain regions, such as ischemic stroke and hemorrhagic stroke (O'Brien and Thomas, 2015). There are more than 50 million people worldwide suffering from dementia, and 33% of all dementia cases are vascular dementia (VD), making it the first most common form of dementia except for Alzheimer's disease (AD) (Smith, 2017). When the complexity of tasks in our daily life increasing, VD patients will have a decline in thinking and cognitive function, moreover, some patients will suffer from mental and emotional abnormalities, including forgetfulness, depression, and anxiety (Kuring et al., 2018). As a result, severe VD will cause serious impact on the quality of patients life and their family members, as well as increase heavy burden on society (Cui et al., 2019). In recent years, with the accelerating of the global population aging, VD has become one of the major public health challenges of the 21st century, which deserves more attention (Grande et al., 2020).

Currently, VD lacks a consensus treatment plan. The most widely used drugs in clinical practice include cholinesterase inhibitors, N-methyl-D-aspartate receptor (NMDA) antagonists, and calcium antagonists (Farooq et al., 2017; Battle et al., 2021). The first two classes of drugs were approved by the US Food and Drug Administration (FDA) for the treatment of AD, mainly including donepezil, rivastigmine, galantamine and memantine. These drugs can only produce a transient relief of cognitive dysfunction in mild, moderate or a few patients with severe VD, and they cannot reverse or cure the disease. Additionally, all of them have side effects, such as diarrhea, nausea, and vomiting (Sun, 2018). Calcium antagonists can selectively act on smooth muscle cells of cerebral blood vessels, effectively eliminate cerebral vascular spasm, dilate blood vessels, and increase blood flow, so they are widely used for the treatment of ischemic neurological injury (Lin et al., 2019; Carlson et al., 2020). However, patients are also subjected to side effects such as blood pressure drop and hepatitis while taking nimodipine tablets (St-Onge et al., 2017). Therefore, green and effective methods for VD treatment and therapy is urgently needed.

Acupuncture is the most widely used traditional and complementary medicine, it was used in 113 countries worldwide, and was commonly performed to treat disorders of the motor, nervous, digestive, endocrine, and reproductive systems (Zhang et al., 2022). Recent studies have found that acupuncture showed very clear efficacy in the treatment of VD (Lu et al., 2022). With the deepening of animal studies, a large amount of evidences showed that acupuncture can effectively treat cognitive dysfunction in animals through multiple targets or pathways (Ye et al., 2017a). Ma et al. (2020) conducted an Magnetic Resonance Imaging (MRI)imaging-based acupuncture study, which found that the combination of GV20 + ST36 significantly attenuated the loss of myelin basic proteins and obviously reduced Interleukin-1β (IL-1β), Interleukin-6 (IL-6), and Ionized calcium bindingadaptor molecule-1 (Iba-1) by improving the perfusion and integrity of white matter. A quantitative proteomic study Isobaric Tags for Relative and Absolute Quantitation (iTRAQ) performed by Yang et al. (2018a) showed that most Differentially Expressed Proteins (DEPs) were associated with oxidative stress, apoptosis, and synaptic function, additionally, the proteins associated with acupuncture effects were also significantly involved in these three cellular processes. The results of this study showed that acupuncture reduced Reactive Oxygen Species (ROS) production, increased neuronal cell survival, and improved Long-term Potential (LTP) in VD rats (Yang et al., 2018a).

As an important body of preclinical evidence, animal experiments play an irreplaceable role in improving the efficiency of clinical treatment. Only a direct comparative study of acupuncture in the treatment of VD has been conducted so far, but many key factors such as acupuncture combinations, acupuncture methods, courses of treatment, and treatment mechanisms have not been involved. Additionally, this study was done 5 years ago (Zhang et al., 2018). Thus, more comprehensive and new studies are needed. In fact, systematic review of animal experiments can increase the probability of successful clinical trials, reduce the producibility of clinical studies, and clarify the underlying mechanisms of acupuncture. In this study, the Bayesian network meta-analysis model was used to comprehensively compare the therapeutic effects of acupuncture, medicine, non-acupoint, and other interventions (Kruschke, 2021). Subgroup analysis was also performed to analyze points, methods and courses of acupuncture. Finally, the mechanism of acupuncture was summarized and sublimated. The conclusions of this research can provide a reference for animal research of acupuncture.

## Materials and methods

### Search strategy

This meta-analysis was conducted according to the PRISMA 2020 statement: an updated guidelines for reporting systematic reviews (Page et al., 2021). Data searching, extraction, and analysis were performed according to our previously published protocol (https://inplasy.com/inplasy-2021-11-0036/; INPLASY2021110036). Two authors (GY Li and YL Shi) independently searched the databases of Pubmed, Embase, Web of science (including Medline). The search time is limited to the establishment of the database until April 2022. The search terms are: acupuncture, electroacupuncture, acupoint, vascular dementia, infarct dementia, vascular cognitive impairment. Each search word are used alone or in combination.

### Inclusion and exclusion criteria

#### Inclusion criteria

Subjects: animals (rats, mice).

Interventions: Acupuncture.

Comparison: Normal group (Gn), Sham-operated group (Gs), Impaired group (Gi), acupuncture group (Ga), Non-acupoint group (Gna), Medicine group (Gm). Among them, Ga can be divided into Manual Acupuncture (MA) and Electroacupuncture (EA) in subgroup analysis.

Outcome: Morris water maze, including primary outcomes: Escape latency of each group in the hidden platform trial (Escape latency), Number of crossing over the former platform location (Number of crossings). Secondary outcomes: Time spent in the target quadrant, Swimming speed to reach the hidden platform in the hidden platform trial (swimming speed).

#### Exclusion criteria

(1) Non-vascular dementia studies.
(2) Non-randomized controlled design of experimental studies on animals.
(3) Non-acupuncture studies.
(4) Studies of acupuncture combined with drug therapy or acupuncture compared to herbal medicine.
(5) Reviews and conference.
(6) The study did not have the outcome of the water maze.
(7) Duplicate and data-identical studies.

### Data extraction

Two authors (YL Shi and L Zhang) independently extracted data from articles that met the inclusion criteria. Extraction included the following: TABLE I: ① Name of first author and year of publication ② animal species, sex, age, weight ③ Modeling method ④ Rest time after modeling ⑤ Interventions ⑥ Drug dose ⑦ Outcomes. TABLE II: ① Name of first author and year of publication ② Acupuncture methods ③ Acupuncture points ④ non-acupoints location ⑤ Time of each acupuncture treatment ⑥ Total course of treatment.

If different frequencies of acupuncture appear in the article, the data with the highest frequency is extracted. When the main data is missing from the included literature or it is presented graphically, we will try to contact the authors to obtain the original data. If the author does not reply, the values in the figure are scanned by the GetData Graph Digitizer 2.26 software (Wang R. et al., 2021).

### Risk of bias

Two investigators (YL Shi and L Zhang) independently assessed the risk of bias for each included study using the SYRCLE's Risk of Bias tool, which included the following: selection bias (sequence generation, baseline characteristics and allocation concealment), performance bias (random housing and blinding), detection bias (random outcome assessment and blinding), attrition bias (incomplete outcome data), reporting bias (selective outcome reporting), other sources of bias (Hooijmans et al., 2014). If disagreements are encountered, they will be resolved through discussions with a third author.

### Statistical analysis

Pair-wise Meta-Analysis: Direct comparisons between interventions were calculated using the Pair-wise Meta-analysis panel of Addis 1.16.8, under the premise of a random effects model based on the D-L method.

Subgroup analysis: Review Manager 5.3 was used to conduct subgroup analysis of different acupoint combinations, acupuncture methods and treatment courses in the acupuncture group, and explore the efficacy differences between the acupuncture group and the impaired group. When heterogeneity occurs, sensitivity analysis is performed to find the source.

Network Meta-Analysis: The results of continuous variables were expressed as mean differences (MD) and 95% CI according to the type of the variable. The network diagrams were drawn using Stata 14, and network meta-analysis was performed using Addis 1.16.8. Addis' model analysis included “consistency” and “inconsistency”. Consistency models can assess the size of effect sizes between interventions and can also calculate the rankings between groups of interventions. When the 95% CI of the results did not contain 0, it indicated that the comparison between interventions was statistically significant (*P* < 0.05).

Node split models: The Node split models is a method for judging whether direct and indirect comparison are consistent. *P* > 0.05, indicating consistency between direct and indirect comparison between interventions, using the consistency model. *P* < 0.05, indicating inconsistency between direct and indirect comparison between interventions, using the inconsistency model. If the outcome indicators cannot be tested for Node split models, the inconsistency model is directly used for analysis. The Potential Scale Reduction Factor (PSRF) evaluates the convergence of the model. If the PSRF value is close to 1, the model has good convergence and the results are stable and reliable. If PSRF < 1.2, it is considered acceptable.

## Results

### Screening process

According to the search strategy set by two researchers, the researchers retrieved a total of 959 literatures from databases. We summarized the retrieved literature and eliminated duplicates, First screen, by reading title and abstract, then delete non-VD, non-randomized controlled animal studies, non-acupuncture, acupuncture combined with other therapy studies, conference papers, reviews. In the second screening, by reading the full text, the research without behavioral indicators, the research on non-water maze indicators and the research on data similarity were excluded, and finally quantitative and qualitative analysis was carried out (Supplementary Figure 1).

### Study characteristics

Fourty-two articles were included in this meta-analysis, including 30 in English (Wang et al., 2004, 2015; Shao et al., 2008; Wei et al., 2011; Zhao et al., 2011; Zhu et al., 2012, 2013, 2018; Feng et al., 2013; Yang et al., 2014, 2018b, 2020; Zhang et al., 2014; Li et al., 2015, 2016; Han et al., 2017; Lin et al., 2017; Liu et al., 2017; Ye et al., 2017b; Du et al., 2018; He et al., 2018; Su et al., 2019; Ma et al., 2020; Wang L. et al., 2020; Wang Z. et al., 2020; Zheng et al., 2020; Cao et al., 2021; Pan et al., 2021; Wang H. L. et al., 2021; Bu et al., 2022) and 12 in Chinese (Li and Lai, 2007; Lin and Wang, 2008; Niu et al., 2009; Tian et al., 2015; Jiang et al., 2017; Zhang et al., 2017; Yang et al., 2019; Gao et al., 2020; Guo et al., 2020; Li et al., 2021; Chen et al., 2022; Xu and Zhang, 2022). The publication year of the article was from June 2004 to April 2022. The research was carried out in China, with a total of 1486 animals. The details are as follows (Supplementary Tables 1, 2).

**Figure 1:**
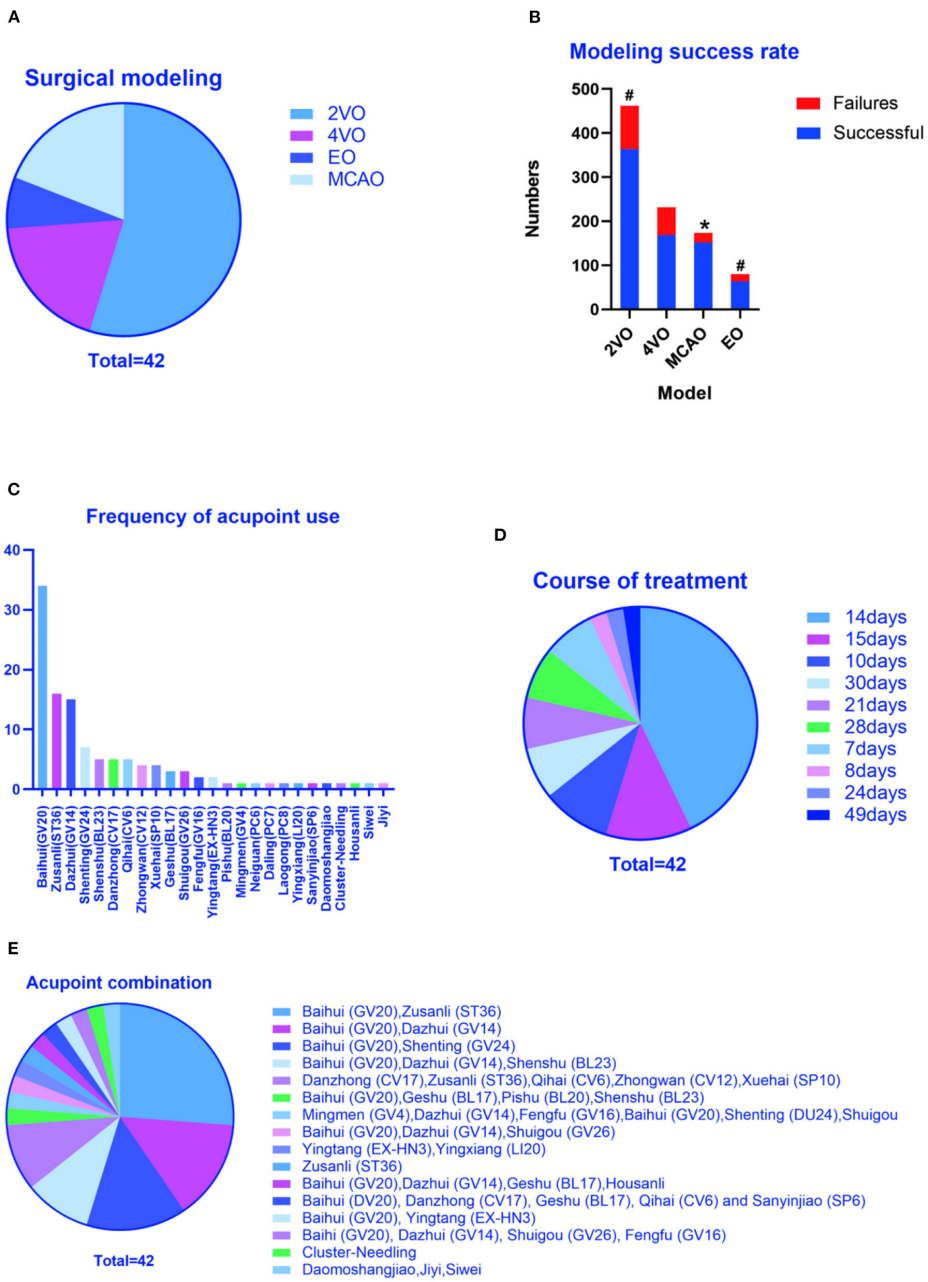
Study characteristics. **(A)** Methods of surgical modeling, **(B)** Total number of modeling and the number of successful modeling, **(C)** Frequency of acupoint use, **(D)** Total course of treatment, **(E)** Frequency of acupoint combination. 2VO, Two - Vessel Occlusion or Bilateral Common Carotid Artery Occlusion; 4VO, Four-Vessel Occlusion; MCAO, Middle Cerebral Artery Occlusion; EO, Embolic occlusion, *P < 0.05 (Modeling success rate of MCAO VS 2VO, EO and 4VO, MCAO had the highest success rate). ^#^*P* < 0.05 (Modeling success rate of 2VO VS 4VO and EO VS 4VO, no difference between 2VO and EO, and the lowest is 4VO).

Methods of surgical modeling: There are 4 surgical modeling methods involved in this study, including 23 articles in the literature using bilateral common carotid artery occlusion (2VO) (Lin and Wang, 2008; Wei et al., 2011; Zhu et al., 2012, 2013, 2018; Yang et al., 2014, 2018b, 2019, 2020; Wang et al., 2015; Li et al., 2016, 2021; Han et al., 2017; Ye et al., 2017b; Gao et al., 2020; Guo et al., 2020; Ma et al., 2020; Wang L. et al., 2020; Cao et al., 2021; Pan et al., 2021; Chen et al., 2022; Xu and Zhang, 2022), 8 articles in the literature using 4-vessel occlusion (4VO) (Wang et al., 2004; Li and Lai, 2007; Shao et al., 2008; Niu et al., 2009; Tian et al., 2015; Jiang et al., 2017; Zhang et al., 2017; Bu et al., 2022), 3 articles in the literature using embolic occlusion (EO) (Zhao et al., 2011; Zhang et al., 2014; Li et al., 2015), and 8 articles in the literature using middle cerebral artery occlusion (MCAO) (Feng et al., 2013; Lin et al., 2017; Liu et al., 2017; He et al., 2018; Su et al., 2019; Wang Z. et al., 2020; Zheng et al., 2020; Wang H. L. et al., 2021). Among them, 24 articles clearly stated the total number of the models and the number of successful models (Wang et al., 2004; Li and Lai, 2007; Lin and Wang, 2008; Shao et al., 2008; Wei et al., 2011; Feng et al., 2013; Zhu et al., 2013; Li et al., 2016, 2021; Han et al., 2017; Jiang et al., 2017; Lin et al., 2017; Liu et al., 2017; Ye et al., 2017b; Zhang et al., 2017; Du et al., 2018; He et al., 2018; Yang et al., 2018b, 2019, 2020; Su et al., 2019; Gao et al., 2020; Guo et al., 2020; Ma et al., 2020; Wang L. et al., 2020; Wang Z. et al., 2020; Zheng et al., 2020; Cao et al., 2021; Wang H. L. et al., 2021; Bu et al., 2022; Chen et al., 2022). According to statistics, MCAO had the highest success rate, no difference between 2VO and EO, and the lowest 4VO (Figures 1A,B).

Interventions: Including normal rats, sham-operated, impaired model, acupuncture, non-acupoint, and medicine.

Outcomes: All literatures have escape latency as an important outcome in the water maze, of which 23 included the number of original platform crossings (Wang et al., 2004; Li and Lai, 2007; Shao et al., 2008; Niu et al., 2009; Wei et al., 2011; Feng et al., 2013; Zhang et al., 2014, 2017; Tian et al., 2015; Jiang et al., 2017; Lin et al., 2017; Liu et al., 2017; He et al., 2018; Su et al., 2019; Yang et al., 2019; Gao et al., 2020; Guo et al., 2020; Ma et al., 2020; Wang Z. et al., 2020; Pan et al., 2021; Wang H. L. et al., 2021; Bu et al., 2022; Xu and Zhang, 2022), 11 literatures (Lin and Wang, 2008; Li et al., 2016; Han et al., 2017; Ye et al., 2017b; Zhu et al., 2018; Ma et al., 2020; Yang et al., 2020; Zheng et al., 2020; Cao et al., 2021; Pan et al., 2021; Xu and Zhang, 2022) calculated the time spent in target quadrant, and 7 literatures (Li et al., 2015; Wang et al., 2015; Liu et al., 2017; Yang et al., 2018b, 2020; Wang L. et al., 2020; Wang Z. et al., 2020) calculated the swimming speed.

Acupuncture methods: two types were included, manual acupuncture (MA) (Zhao et al., 2011; Yang et al., 2014, 2018b, 2019, 2020; Li et al., 2015, 2016, 2021; Tian et al., 2015; Wang et al., 2015; Ye et al., 2017b; Du et al., 2018; Zhu et al., 2018; Su et al., 2019; Gao et al., 2020; Ma et al., 2020; Wang L. et al., 2020; Cao et al., 2021; Pan et al., 2021; Xu and Zhang, 2022) and electroacupuncture (EA) (Wang et al., 2004; Li and Lai, 2007; Lin and Wang, 2008; Shao et al., 2008; Niu et al., 2009; Wei et al., 2011; Zhu et al., 2012, 2013; Feng et al., 2013; Han et al., 2017; Jiang et al., 2017; Liu et al., 2017; Zhang et al., 2017; He et al., 2018; Guo et al., 2020; Wang Z. et al., 2020; Zheng et al., 2020; Wang H. L. et al., 2021; Bu et al., 2022; Chen et al., 2022).

Frequency of acupoint use and frequency of acupoint combination: All literature clearly pointed out acupuncture points. According to statistics, Baihui (GV20) (Wang et al., 2004, 2015; Li and Lai, 2007; Lin and Wang, 2008; Shao et al., 2008; Wei et al., 2011; Zhu et al., 2012, 2013, 2018; Feng et al., 2013; Li et al., 2016, 2021; Han et al., 2017; Jiang et al., 2017; Lin et al., 2017; Liu et al., 2017; Ye et al., 2017b; Zhang et al., 2017; Du et al., 2018; He et al., 2018; Yang et al., 2018b, 2019, 2020; Su et al., 2019; Gao et al., 2020; Guo et al., 2020; Ma et al., 2020; Wang L. et al., 2020; Wang Z. et al., 2020; Zheng et al., 2020; Cao et al., 2021; Bu et al., 2022; Chen et al., 2022), bilateral Zusanli (ST36) (Zhao et al., 2011; Zhang et al., 2014; Li et al., 2015, 2016, 2021; Wang et al., 2015; Ye et al., 2017b; Du et al., 2018; Yang et al., 2018b, 2020; Zhu et al., 2018; Ma et al., 2020; Wang L. et al., 2020; Cao et al., 2021; Pan et al., 2021; Xu and Zhang, 2022) and Dazhui (GV14) (Wang et al., 2004; Li and Lai, 2007; Lin and Wang, 2008; Shao et al., 2008; Wei et al., 2011; Zhu et al., 2012, 2013; Han et al., 2017; Jiang et al., 2017; Zhang et al., 2017; Su et al., 2019; Yang et al., 2019; Gao et al., 2020; Guo et al., 2020; Chen et al., 2022) were used the most frequently (Figure 1C). According to the analysis of the acupoint combinations in the article, the combination of Baihui + bilateral Zusanli (GV20+ST36) (Wang et al., 2015; Li et al., 2016, 2021; Ye et al., 2017b; Du et al., 2018; Yang et al., 2018b, 2020; Zhu et al., 2018; Ma et al., 2020; Wang L. et al., 2020; Cao et al., 2021), Baihui + Dazhui (GV20+GV14) (Wang et al., 2004; Li and Lai, 2007; Wei et al., 2011; Han et al., 2017; Jiang et al., 2017; Zhang et al., 2017) and Baihui + Shenting (GV20+GV24) (Feng et al., 2013; Lin et al., 2017; Liu et al., 2017; He et al., 2018; Wang Z. et al., 2020; Wang H. L. et al., 2021) is the most frequently used, respectively 26.19, 14.29, and 14.29% (Figure 1E).

Non-acupoint: Fifteen studies in the literature used sham acupuncture or non-acupoint studies. eight of them (Zhao et al., 2011; Li et al., 2015, 2016, 2021; Ye et al., 2017b; Du et al., 2018; Yang et al., 2018b; Ma et al., 2020) selected in the bilateral hypochondrium, 10 mm above the iliac crest as a sham acupuncture point, three of them (Wang et al., 2015; Zhu et al., 2018; Wang Z. et al., 2020) selected 2 cm higher to the anterior superior spine. Two article (Zhang et al., 2014; Pan et al., 2021) 2 articles selected in the hypochondrium, 3 mm above the iliac crest. One article (Lin and Wang, 2008) selected at the thoracoabdominal junction of the first and second lumbar vertebrae. One article (Bu et al., 2022) located on the meridian route, and did not belong to the traditional 14 meridians.

The time of each acupuncture treatment and the total course of treatment: 41 literatures specified the time of each acupuncture treatment, and 1 literature (Li et al., 2021) did not specify. The main time is 30 min (31.71%) (Feng et al., 2013; Tian et al., 2015; Han et al., 2017; Jiang et al., 2017; Lin et al., 2017; Liu et al., 2017; He et al., 2018; Yang et al., 2019; Gao et al., 2020; Wang Z. et al., 2020; Wang H. L. et al., 2021; Bu et al., 2022; Chen et al., 2022), 20 min (21.95%) (Wang et al., 2004; Li and Lai, 2007; Lin and Wang, 2008; Shao et al., 2008; Wei et al., 2011; Zhu et al., 2012, 2013; Zhang et al., 2017; Su et al., 2019), 30 S (21.95%) (Zhao et al., 2011; Zhang et al., 2014; Li et al., 2015, 2016; Ye et al., 2017b; Du et al., 2018; Yang et al., 2018b; Pan et al., 2021; Xu and Zhang, 2022) and 10 min (17.07%) (Niu et al., 2009; Zhu et al., 2018; Wang L. et al., 2020; Yang et al., 2020; Zheng et al., 2020; Cao et al., 2021). All literatures indicated the duration of the entire treatment course, namely 14 days (42.86%) (Li et al., 2015, 2016; Wang et al., 2015; Ye et al., 2017b; Du et al., 2018; Yang et al., 2018b, 2019, 2020; Zhu et al., 2018; Gao et al., 2020; Guo et al., 2020; Ma et al., 2020; Wang L. et al., 2020; Wang Z. et al., 2020; Zheng et al., 2020; Cao et al., 2021; Pan et al., 2021; Lu et al., 2022), 15 days (11.90%) (Wang et al., 2004; Li and Lai, 2007; Shao et al., 2008; Su et al., 2019; Xu and Zhang, 2022), 10 days (9.52%) (Wei et al., 2011; Feng et al., 2013; Tian et al., 2015; Jiang et al., 2017) (Figure 1D).

### Risk of bias

The overall quality of the included literatures was medium. According to the SYRCLE risk assessment tool, the evaluation results were as follows: all the literatures mentioned random allocation, of which 8 literatures (Li and Lai, 2007; Lin and Wang, 2008; Jiang et al., 2017; Zhang et al., 2017; Yang et al., 2019; Gao et al., 2020; Guo et al., 2020; Ma et al., 2020) clearly informed the randomization method. All the papers are balanced at baseline, but none of them mentioned allocation concealment. None of the studies indicated whether the animals were randomly housed, but 36 papers specified the housing environment, and the remaining 6 papers unspecified (Wang et al., 2004; Li and Lai, 2007; Lin and Wang, 2008; Niu et al., 2009; Zhu et al., 2013; Su et al., 2019). This study was an acupuncture study, the operator could not be blinded during treatment. All animals in the 30 studies were evaluated for outcome evaluation, but 12 studies (Lin and Wang, 2008; Shao et al., 2008; Niu et al., 2009; Zhu et al., 2012; Zhang et al., 2014; Li et al., 2016; Han et al., 2017; Liu et al., 2017; Ye et al., 2017b; Wang L. et al., 2020; Wang Z. et al., 2020; Yang et al., 2020) selected some animals for evaluation without specifying whether they were randomly selected. In terms of outcome statistics, 2 articles in the literature explicitly mentioned blinding (Li et al., 2015; Cao et al., 2021), 2 articles in the literature (Shao et al., 2008; Zhu et al., 2013) were counted by the first author, and the remaining 38 articles did not mention statistical blinding. The results of the data for the entire literatures were completed and no selective reporting was evaluated. In terms of other biases, 19 articles in the literature (Li and Lai, 2007; Lin and Wang, 2008; Zhao et al., 2011; Yang et al., 2014; Zhang et al., 2014, 2017; Tian et al., 2015; Jiang et al., 2017; Liu et al., 2017; He et al., 2018; Su et al., 2019; Gao et al., 2020; Ma et al., 2020; Wang Z. et al., 2020; Li et al., 2021; Pan et al., 2021; Wang H. L. et al., 2021; Bu et al., 2022; Chen et al., 2022) had no other biases, and the remaining 23 (Wang et al., 2004, 2015; Shao et al., 2008; Niu et al., 2009; Wei et al., 2011; Zhu et al., 2012, 2013, 2018; Feng et al., 2013; Li et al., 2015, 2016; Han et al., 2017; Lin et al., 2017; Ye et al., 2017b; Du et al., 2018; Yang et al., 2018b, 2019, 2020; Su et al., 2019; Guo et al., 2020; Wang L. et al., 2020; Cao et al., 2021; Xu and Zhang, 2022) did not mention the number of successful surgical modeling or the rest time after modeling (Supplementary Figures 2, 3).

### Pair-wise meta-analysis

We compared the escape latency, the number of crossing, and the time spent in the target quadrant. some of the results were the same: Ga was better than Gi and Gna, Ga was worse than Gs and Gn, Gi was worse than Gs and Gn, and Gna was worse than Gs. We compared the swimming speed of VD rats, except that Gn was significant compared to Gna, the rest of the comparisons did not reach the level of significance (Supplementary Table 3).

### Subgroup analysis

Escape latency is the most important indicator in the water maze. We performed subgroup analysis on this outcome according to different acupoint combinations, acupuncture methods, and treatment courses.

Escape latency: The statistical results of 42 studies showed that Ga could significantly shorten the escape latency compared to Gi (MD: −25.78; 95%CI: −29.20, −22.37; I^2^ = 96%). Subgroup analysis was performed according to different acupoint combinations, acupuncture methods and courses of treatment, GV20 + ST36 (MA) (Wang et al., 2015; Li et al., 2016, 2021; Ye et al., 2017b; Du et al., 2018; Yang et al., 2018b, 2020; Zhu et al., 2018; Ma et al., 2020; Wang L. et al., 2020; Cao et al., 2021) (MD: −23.41; 95% CI: −26.66, −20.16; I^2^ = 0%), no heterogeneity within the group. GV20 + GV14 (EA) (Wang et al., 2004; Li and Lai, 2007; Wei et al., 2011; Han et al., 2017; Jiang et al., 2017; Zhang et al., 2017) (MD: −27.54; 95%CI: −36.29, −18.79; I^2^ = 97%), with greater heterogeneity within the group. GV20+GV24 (EA) (Feng et al., 2013; Lin et al., 2017; Liu et al., 2017; He et al., 2018; Wang Z. et al., 2020; Wang H. L. et al., 2021) (MD: −24.18; 95%CI: −30.49, −17.87; I^2^ = 86%), with greater heterogeneity within the group. GV20 + GV14 + BL23 (EA) (Lin and Wang, 2008; Zhu et al., 2012, 2013; Chen et al., 2022) (MD: −13.26; 95%CI: −16.82, −9.70; I^2^ = 3%), no heterogeneity within the group. CV6 + CV12 + CV17 + ST36 + SP10 (MA) (Zhao et al., 2011; Zhang et al., 2014; Pan et al., 2021; Xu and Zhang, 2022) (MD: −9.99; 95%CI: −13.98, −6.00; I^2^ = 75%), with mediun heterogeneity within the group. Other Acupoints (MA or EA) (Shao et al., 2008; Niu et al., 2009; Yang et al., 2014, 2019; Li et al., 2015; Tian et al., 2015; He et al., 2018; Su et al., 2019; Gao et al., 2020; Guo et al., 2020; Zheng et al., 2020; Bu et al., 2022) (MD: −39.62; 95%CI: −51.05, −28.19; I^2^ = 97%), with greater heterogeneity within the group (Supplementary Figure 4).

Sensitivity analysis was performed for the heterogeneity within the group of GV20 + GV14 (EA) (Wang et al., 2004; Li and Lai, 2007; Wei et al., 2011; Han et al., 2017; Jiang et al., 2017; Zhang et al., 2017) and Other Acupoints (MA or EA) (Shao et al., 2008; Niu et al., 2009; Yang et al., 2014, 2019; Li et al., 2015; Tian et al., 2015; He et al., 2018; Su et al., 2019; Gao et al., 2020; Guo et al., 2020; Zheng et al., 2020; Bu et al., 2022). Deletion of any study did not remove heterogeneity, but the results were still the same as before, the reason for the heterogeneity may be the course of treatment caused by inconsistency. The heterogeneity of GV20 + GV24 (EA) (Feng et al., 2013; Lin et al., 2017; Liu et al., 2017; He et al., 2018; Wang Z. et al., 2020; Wang H. L. et al., 2021) was caused by He et al. (2018), whose postoperative rest period was 7 days, which was longer than the rest of the literature. The heterogeneity of CV6 + CV12 + CV17 + ST36 + SP10 (MA) (Zhao et al., 2011; Zhang et al., 2014; Pan et al., 2021; Xu and Zhang, 2022) was caused by Zhao et al. (2011) or Xu and Zhang (2022), and their treatments were different. Statistical results showed that GV20 + ST36 (MA) (Wang et al., 2015; Li et al., 2016, 2021; Ye et al., 2017b; Du et al., 2018; Yang et al., 2018b, 2020; Zhu et al., 2018; Ma et al., 2020; Wang L. et al., 2020; Cao et al., 2021) was better than other subgroups in terms of heterogeneity and Z value. The duration of this combination of treatment was 14 days, and MA was used uniformly as an acupuncture method.

### Network meta-analysis results

**Figure 2:**
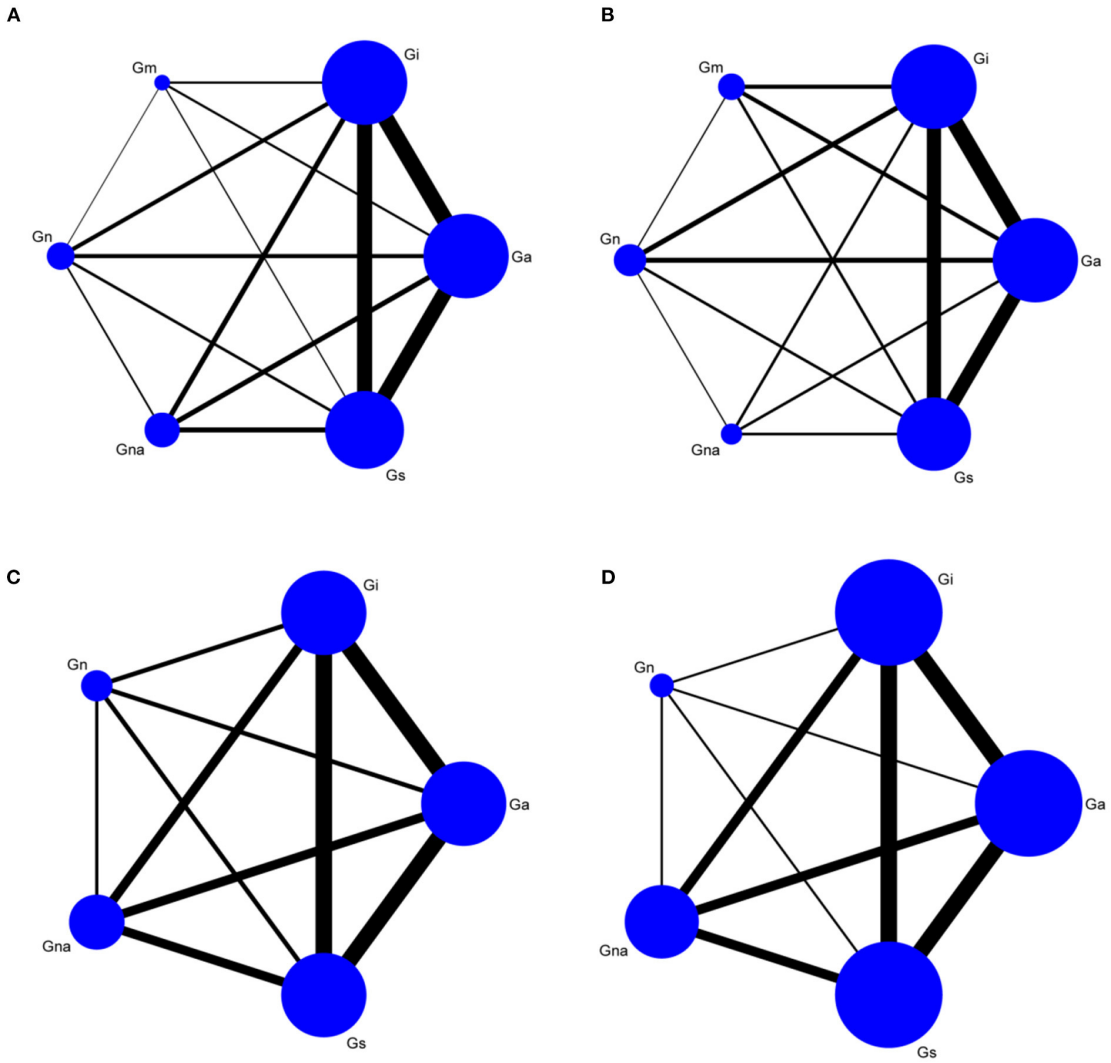
Network graph of morris water maze. Escape latency **(A)**, Number of crossings **(B)**, Time spent in target quadrant **(C)**, Swimming speed **(D)**. Gn, Normal group; Gs, Sham-operated group; Gi, Impaired group; Ga, acupuncture group; Gna, Non-acupoint group; Gm, Medicine group.

We performed a network meta-analysis of escape latency, number of crossings, time spent in target quadrant, and swimming speed under various interventions (Figure 2). Escape latency, number of original crossings, and the time spent in target were tested using the node-split model to evaluate the consistency between direct and indirect comparisons. The results showed that *P* > 0.05, PSRF = 1.0, and the results were analyzed using the consistency model (Supplementary Figure 5). The Node Split model cannot be used to test swimming speed. To ensure the objectivity of the results, the inconsistency model is used directly for analysis, and the PSRF value is 1.0. The results are stable and reliable.

#### Escape latency

Forty-two studies with a total of 1,486 animals, 6 interventions framed NMA for escape latency, and the results were as follows: Ga was superior to Gi and Gna, and Ga was inferior to Gn and Gs. Gi was inferior to Gm, Gn, Gna, and Gs. Gm was better than Gna. Gn was better than Gna. Gna was less than Gs. P < 0.05 for all the above results (Table 1A).

**Table 1:** Network meta-analysis of MWM.

**(A) Escape latency**					
Ga					
−25.87 (−30.75, −21.12)	Gi				
4.35 (−6.32, 14.48)	30.09 (19.58, 40.46)	Gm			
13.73 (5.76, 21.88)	39.58 (31.44, 47.53)	9.60 (−2.51, 21.54)	Gn		
−15.73 (−23.30, −8.15)	10.16 (2.59, 17.66)	−19.99 (−32.00, −7.53)	−29.40 (−39.26, −19.82)	Gna	
6.79 (1.92, 11.69)	32.68 (27.80, 37.61)	2.58 (−7.88, 13.19)	−6.92 (−15.18, 1.34)	22.54 (15.14, 29.90)	Gs
**(B) Number of crossings**					
Ga					
2.63 (1.94, 3.34)	Gi				
0.09 (−1.09, 1.32)	−2.54 (−3.73, −1.32)	Gm			
−1.29 (−2.41, −0.17)	−3.91 (−5.05, −2.81)	−1.39 (−2.87, 0.09)	Gn		
1.03 (−0.36, 2.41)	−1.61 (−3.01, −0.21)	0.93 (−0.80, 2.65)	2.32 (0.70, 3.92)	Gna	
−1.31 (−2.04, −0.57)	−3.95 (−4.69, −3.20)	−1.41 (−2.70, −0.18)	−0.03 (−1.22, 1.15)	−2.34 (−3.75, −0.93)	Gs

**(C) Time spent in the target quadrant**				
Ga				
7.92 (4.45, 11.48)	Gi			
−8.42 (−14.52, −2.65)	−16.35 (−22.26, −10.64)	Gn		
7.68 (3.35, 12.23)	−0.25 (−4.54, 4.18)	16.13 (9.79, 22.44)	Gna	
−5.36 (−9.03, −1.74)	−13.29 (−16.88, −9.69)	3.09 (−2.80, 9.03)	−13.03 (−17.48, −8.53)	Gs
**(D) Swimming speed**				
Ga				
0.20 (−1.22, 1.71)	Gi			
1.87 (−0.76, 4.24)	1.58 (−0.75, 4.07)	Gn		
0.11 (−1.38, 1.52)	−0.34 (−1.90, 1.06)	−2.08 (−4.55, 0.42)	Gna	
0.24 (−1.51, 1.81)	−0.05 (−1.65, 1.52)	−1.74 (−4.66, 0.55)	0.18 (−1.35, 1.82)	Gs

*
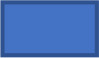
Treatments, 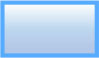
Efficacy (MD [95% Crl]), 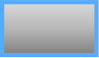
P < 0.05; Efficacy (MD [95% Crl]), P > 0.05. Gn, Normal group; Gs, Sham-operated group; Gi, Impaired group; Ga, acupuncture group; Gna, Non-acupoint group; Gm. Medicine group*.

**Figure 3:**
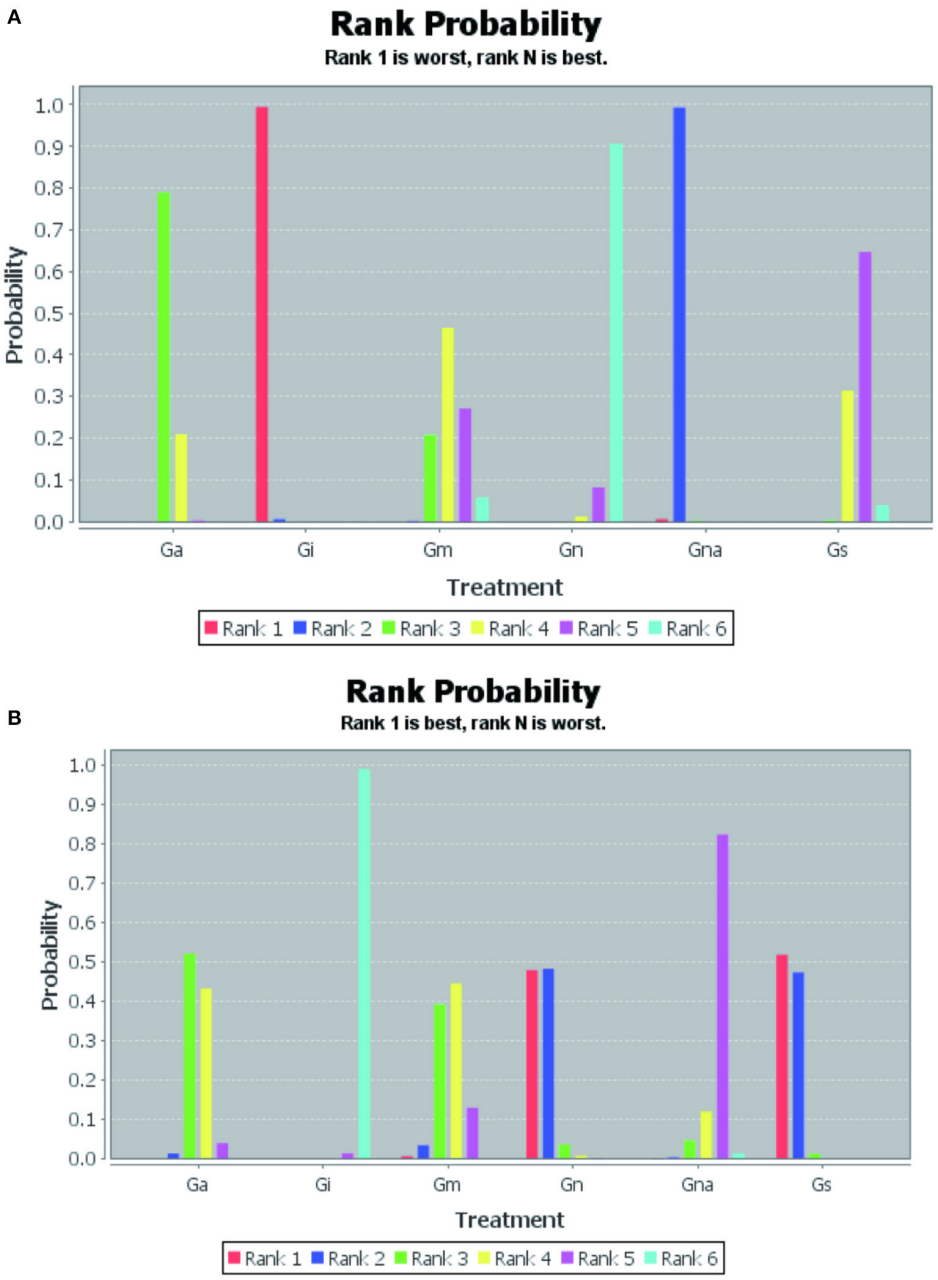
Rank probability of interventions. Escape latency **(A)**, Number of crossings **(B)**. Gn, Normal group; Gs, Sham-operated group; Gi, Impaired group; Ga, acupuncture group; Gna, Non-acupoint group; Gm, Medicine group.

The probability of escape latency in the treatment of VD under multiple interventions was ranked as follows: Gn (90%), Gs (65%), Gm (46%), Ga (78%), Gna (99%), Gi (99%) (Figure 3A).

#### Number of crossing

Twenty-three studies (Wang et al., 2004; Li and Lai, 2007; Shao et al., 2008; Niu et al., 2009; Wei et al., 2011; Feng et al., 2013; Zhang et al., 2014, 2017; Tian et al., 2015; Jiang et al., 2017; Liu et al., 2017; He et al., 2018; Su et al., 2019; Yang et al., 2019; Gao et al., 2020; Guo et al., 2020; Ma et al., 2020; Wang Z. et al., 2020; Pan et al., 2021; Wang H. L. et al., 2021; Bu et al., 2022; Xu and Zhang, 2022) with a total of 848 animals and 6 interventions fromed the NMA with the following results: Ga was superior to Gi and Ga was inferior to Gn and Gs. Gi was less than Gm, Gn, Gna, and Gs. Gm was less than Gs. Gna was less than Gs. *P* < 0.05 for all the above results (Table 1B).

The original platform crossing times for the treatment of VD under various interventions were ranked from superior to inferior probability: Gs (52%), Gn (48%), Ga (52%), Gm (44%), Gna (82%), Gi (99%) (Figure 3B).

#### Time spent in the target quadrant

Eleven studies (Lin and Wang, 2008; Li et al., 2016; Han et al., 2017; Ye et al., 2017b; Zhu et al., 2018; Ma et al., 2020; Yang et al., 2020; Zheng et al., 2020; Cao et al., 2021; Pan et al., 2021; Xu and Zhang, 2022) with a total of 377 animals and five interventions formed the NMA and the results were as follows: Ga was superior to Gi and Gna, and Ga was inferior to Gn and Gs. Gi was inferior to Gn and Gs. Gn was better than Gna. Gna was less than Gs. *P* < 0.05 for all the above results (Table 1C).

The time spent in the target quadrant for the treatment of VD under various interventions were ranked from superior to inferior probability: Gn (85%), Gs (85%), Ga (99%), Gna (55%), Gi (55%) (Supplementary Figure 6).

#### Swimming speed

Seven studies (Li et al., 2015; Wang et al., 2015; Liu et al., 2017; Yang et al., 2018b, 2020; Wang L. et al., 2020; Wang Z. et al., 2020) with a total of 252 animals, 5 interventions formed the NMA, the results of the network meta-analysis of swimming speed are shown in the table, there is no difference between Ga, Gi, Gn, Gna, and Gs (Table 1D).

### Potential mechanism of acupuncture improving cognitive dysfunction in VD rats

Acupuncture improves cognitive dysfunction in VD rats with multi-target characteristics, mainly in: reducing oxidative stress (Wang et al., 2004, 2015; Zhu et al., 2013; Zhang et al., 2014; Li et al., 2016, 2021; Du et al., 2018; Yang et al., 2018b; Su et al., 2019), neuronal inflammation (Li and Lai, 2007; Han et al., 2017; Liu et al., 2017; Ma et al., 2020; Wang L. et al., 2020; Cao et al., 2021; Pan et al., 2021; Bu et al., 2022; Chen et al., 2022; Xu and Zhang, 2022), and apoptosis (Feng et al., 2013; Tian et al., 2015; Zhang et al., 2017; Zhu et al., 2018; Guo et al., 2020; Wang H. L. et al., 2021), increasing synaptic plasticity (Wei et al., 2011; Zhu et al., 2012; Lin et al., 2017; Ye et al., 2017b; Wang Z. et al., 2020; Zheng et al., 2020), neurotransmitter (Lin and Wang, 2008; Shao et al., 2008; Wei et al., 2011; Yang et al., 2014, 2019; Jiang et al., 2017; Ye et al., 2017b; He et al., 2018), and neuron numbers (Li et al., 2015), improving Vascular function (Gao et al., 2020) and glucose metabolism (Zhao et al., 2011), etc. One article (Yang et al., 2020) in all the studies explored the differences in the efficacy of different acupuncture frequencies, but no specific mechanism was involved.

## Discussion

### Main findings guiding animal experiments

Data for this meta-analysis came from 42 animal studies of acupuncture in the treatment of vascular dementia. The included studies were carried out with reference to the control design, with a total of 1,486 animals. We evaluated the included literature with reference to the SYRCLE animal research evaluation criteria (Hooijmans et al., 2014), and the results were that 17 (Zhao et al., 2011; Yang et al., 2014; Li et al., 2015, 2021; Tian et al., 2015; Jiang et al., 2017; Liu et al., 2017; Zhang et al., 2017; He et al., 2018; Gao et al., 2020; Ma et al., 2020; Zheng et al., 2020; Cao et al., 2021; Pan et al., 2021; Wang H. L. et al., 2021; Bu et al., 2022; Chen et al., 2022) quality were good, 22 (Wang et al., 2004, 2015; Li and Lai, 2007; Lin and Wang, 2008; Wei et al., 2011; Zhu et al., 2012, 2018; Feng et al., 2013; Zhang et al., 2014; Li et al., 2016; Han et al., 2017; Lin et al., 2017; Ye et al., 2017b; Du et al., 2018; Yang et al., 2018b, 2019, 2020; Su et al., 2019; Guo et al., 2020; Wang Z. et al., 2020; Xu and Zhang, 2022) were moderate, and 3 (Shao et al., 2008; Niu et al., 2009; Zhu et al., 2013) were of low quality. We analyze the characteristics of the included literature. This study includes 4 surgical modeling methods: 2VO, 4VO, EO, and MCAO. This study found that the success rate of MCAO surgical modeling was higher than that of 2VO, EO and 4VO, but different surgical methods could create VD models. Animals have different rest periods after surgery, with a minimum 2 h (Feng et al., 2013; Wang H. L. et al., 2021), which we think may have an impact on the recovery of the animal's body. The duration of treatment in the included literature is different, of which 14 days (Li et al., 2015, 2016; Wang et al., 2015; Ye et al., 2017b; Du et al., 2018; Yang et al., 2018b, 2019, 2020; Zhu et al., 2018; Gao et al., 2020; Guo et al., 2020; Ma et al., 2020; Wang L. et al., 2020; Wang Z. et al., 2020; Zheng et al., 2020; Cao et al., 2021; Pan et al., 2021; Lu et al., 2022) occupy the most, followed by 15 days (Wang et al., 2004; Li and Lai, 2007; Shao et al., 2008; Su et al., 2019; Xu and Zhang, 2022) and 10 days (Wei et al., 2011; Feng et al., 2013; Tian et al., 2015; Jiang et al., 2017). Jiang et al. counts the effects of different treatment days on the curative effect (Jiang et al., 2017). We also believe that the duration of treatment is also one of the important factors to consider.

The results of the network and pair-wise meta-analysis were highly consistent, that is, in terms of escape latency and the number of original platform crossings, Gn and Gs have the best curative effect, followed by Ga and Gm (no significant difference between the them), and finally Gna and Gi. In improving the time spent in the target quadrant, Gn and Gs had the best effect, followed by Ga and finally Gna and Gi. In terms of swimming speed, there were no significant differences between almost all groups.

A small part of the results we have obtained are somewhat different between direct comparison and indirect comparison. After careful investigation, we found that the difference in outcomes was mainly due to the Gna intervention. Therefore, we think that the specific location of Gna needs to be carefully selected, and maybe Gna also has some therapeutic effects, which may be one of the reasons for the difference.

### The rats in the normal group and the sham-operated group had the best cognitive function

The rats in the normal group not received any intervention. The rats in the sham operation group were operated with the separation of the carotid artery and vagus nerve after the skin of the neck was incised and the carotid artery was not ligated. There were no significant differences in the performance of the morris water maze between the two groups of rats, so the absence of carotid artery ligation did not affect the cognitive ability of the rats. The measurement of the water maze reflects mainly the ability of learning and memory, and the anatomical positions related to these abilities are located primarily in the hippocampus and cortex (Washida et al., 2019).

### Acupuncture can significantly improve the cognition in VD rats

Acupuncture has been widely used in the fields of nervous system, digestive system, and rehabilitation treatment. It is one of the important alternative therapies today (Lu et al., 2022). Acupuncture plays an important role in the treatment of vascular dementia. The acupuncture points, methods, and courses of treatment were considered ad the important factors for the efficacy of acupuncture. This study conducted a subgroup analysis of these three factors. The results from the subgroup analysis of acupuncture points selection indicated that the heterogeneity and effective size of the combination of GV20 + ST36 (Baihui+Zusanli, MA, 14 days) (Wang et al., 2015; Li et al., 2016, 2021; Ye et al., 2017b; Du et al., 2018; Yang et al., 2018b, 2020; Zhu et al., 2018; Ma et al., 2020; Wang L. et al., 2020; Cao et al., 2021) were better than those of other combinations of acupuncture points. It has been found that GV20 could regulate cerebral blood flow in the ischemic region and promote nerve regeneration in the central nervous system (Chavez et al., 2017). Electroacupuncture in ST36 can activate the spinal sympathetic reflex and drive the vagal-adrenal axis through nerve endings to achieve anti-inflammatory effects (Liu et al., 2021). In the literatures we included, Ye et al. (2017b) conducted a study on the difference of three combinations efficacy, such as GV20 + ST36, GV20 + GV24, and ST36 + SP10, and the results showed that GV20 + ST36 performed better than other combinations in the detection of the morris water maze. The reason why GV20 + ST36 has better efficacy than other combinations is considered to be that GV20 is an important acupuncture point in the head. During oblique puncturing, the brain tissue stimulated by the needle tip is an important regulatory center for rat behavior, through which signals of acupuncture stimulation can be transmitted to the meninges, brainstem nucleus, or hippocampus. ST36 belongs to the acupuncture points of the hindlimb, with riched muscles in the lower extremities and more sensitive nerve conduction than in the chest and abdomen. Combining the acupuncture points at the upper and lower ends can better send signals back to the brain, thus increasing cerebral blood flow and improving cognition in rats. Yang et al. (2020) conducted a study using different treatment frequencies and stimulation amounts, and the results showed that high stimulation amounts could promote the recovery of cognitive ability in rats. compared to the rats received low stimulation amounts. In the literatures included in this study, GV20 + ST36 basically stimulated for consecutive 30s, with Angle < 90, Frequency > 120. However, the choice of wave type and frequency of electroacupuncture acupoint combination treatment were inconsistent, and the current is basically 1–3 mA, plus the inconsistent course of treatment, resulting in bias. Therefore, we recommend the use of the combination of GV20 + ST36 manual acupuncture with a higher frequency of treatment when conducting animal research on acupuncture for VD.

**Figure 4:**
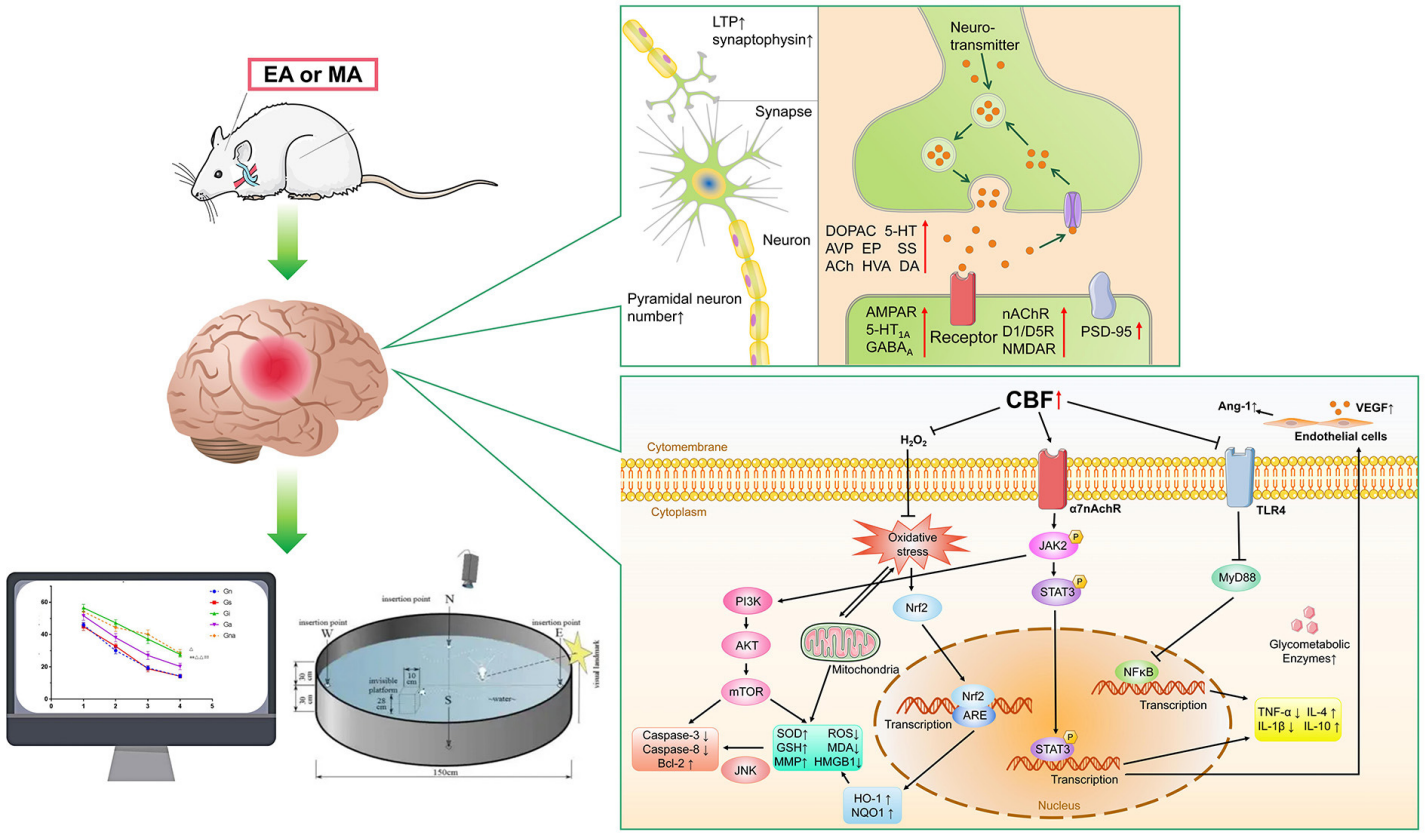
The main mechanism of acupuncture in animal models of VD. EA, electroacupuncture; MA, manual acupuncture. Synapse function: LTP (Long-term Potential), AVP (arginine vasopressin), β-EP (β-endorphine), SS (somatostatin), ACh (acetylcholine), HVA (homovanillic acid), DA (dopamine), DOPAC (Dihydroxyphenylaceticacid), 5-HT (5-hydroxytryptamine),EP (Epinephrine), nAChR (Nicotinic-Acetylcholine receptor), D1/D5R (dopamine 1/ dopamine5 receptor), NMDAR (N-methyl-D-aspartate receptor), AMPAR (α-amino-3-hydroxy-5-methyl-4-isoxazole propionic acid receptor), GABA_A_R (γ-amino-butyric acid type A receptor), 5-HT_1A_R (postsynaptic serotonin (1A) receptors), PSD-95 (postsynaptic density-95). signal pathways: CBF (cerebral blood flow), Ang-1 (Angiopoietin-1), VEGF (vascular endothelial growth factor), TLR4 (Toll Like Receptor 4), MyD88 (myeloid differentiation factor 88), NF-κB (nuclear factor-kappa B), α7nAChR (alpha-7 nicotinic acetylcholine receptor), JAK2 (Janus Kinase 2), STAT3 (Signal Transducer and Activator of Transcription 3), TNF-α (Tumor Necrosis Factor-α), IL-4 (interleukin 4), IL-10 (interleukin 10), IL-1β (interleukin 1β), Nrf2 (Nuclear factor erythroid2-related factor2), ARE (antioxidant response element), HO-1 (heme oxygenase), NQO1 (NADP (H) quinone oxidoreductase), ROS (reactive oxygen species), MDA (Malon-dialdehyde), SOD (superoxide dismutase), GSH (glutathione), HMGB1 (mobility group protein B1), MMP (mitochondrial membrane potential), PI3K (phosphatidylinositol 3-kinase), AKT (protein kinase B), mTOR (mammalian target of rapamycin), Bcl-2 (B-cell lymphoma-2), JNK (c-Jun N-terminal kinase).

In conclusion, this study showed that acupuncture has a clear neuroprotective effect in VD animal models. The mechanism of acupuncture on VD is summarized as follows: (1) Reduction of the oxidative stress (Wang et al., 2004, 2015; Zhu et al., 2013; Zhang et al., 2014; Li et al., 2016, 2021; Du et al., 2018; Yang et al., 2018b; Su et al., 2019), The oxidative stress which caused by chronic cerebrospinal hypoperfusion (CCH) was considered as a major factor in the underlying mechanism of VD, and brain damage induced by oxidative stress is a key step leading to cognitive deficits (Han et al., 2020). Acupuncture can reverse mitochondrial damage and reduce ROS, MDA and HMGB1, increase MMP, SOD, GSH, up-regulate ChAT and 14-3-3e, and down-regulate AChE and S100B activity. This series of effects were mainly caused by the activation of PI3K/AKT and Nrf2/HO-1 pathways. (2) Reduction of neuronal inflammation (Li and Lai, 2007; Han et al., 2017; Liu et al., 2017; Ma et al., 2020; Wang L. et al., 2020; Cao et al., 2021; Pan et al., 2021; Bu et al., 2022; Chen et al., 2022; Xu and Zhang, 2022). Cellular inflammation is one of the most important pathological mechanisms of VD, and is also involved in the development of other pathological mechanisms (Zhang et al., 2020). Acupuncture decreased IL-1β, IL-2, IL-6, TNF-α, INF-γ, MIP-2, iNOS, COX-2, Iba-1 levels and increased IL-4, IL-10 levels through α7nAChR/JAK2/STAT3 or TLR4/MyD88/NF-kB pathways. (3) Reduced apoptosis (Feng et al., 2013; Tian et al., 2015; Zhang et al., 2017; Zhu et al., 2018; Guo et al., 2020; Wang H. L. et al., 2021). Chronic cerebral hypoperfusion induces the expression and activation of inflammatory vesicle components in different regions of the brain and promotes the activation of apoptotic pathways. Cell survival requires regulation by a series of proteins associated with apoptosis (Poh et al., 2021). Acupuncture improved the cognition in VD rats by initiating the PI3K/AKT/mTOR or Trx-1/P-ASK/P-P38/P-JNK channel, increasing the expression of Bcl-2 protein, decreasing the protein levels of BAX, FAX, P53, Caspase 3, Caspase 8, and Aβ1-40. (4) Increased neurotransmitters (Lin and Wang, 2008; Shao et al., 2008; Niu et al., 2009; Yang et al., 2014, 2019; Jiang et al., 2017; He et al., 2018). Brain neurotransmitters are chemicals that help transmit signals from one nerve cell to another. Neurotransmitters are part of the core mechanism expressed by all neurons, and their content is closely related to cognitive ability (Spitzer, 2012). Acupuncture stimulations can increase the secretion of DOPAC, 5-HT, AVP, EP, SS, ACh, HVA, DA and some neurochemicals in VD rats, thereby increasing the level of neurotransmitter release. (5) Increased synaptic plasticity (Wei et al., 2011; Zhu et al., 2012; Lin et al., 2017; Ye et al., 2017b; Wang Z. et al., 2020; Zheng et al., 2020). Synaptic plasticity is a property of the tunable strength of connections between nerve cells, and synaptic plasticity has long been recognized as the neurobiological basis of cognition (Alkadhi, 2021). As an important determinant of synaptic plasticity, LTP plays a critical role in memory formation (Ye et al., 2017b). Acupuncture not only increased synaptic plasticity by upregulating LTP and increasing the amount of synaptophysin, but also increased the nACh, D1/D5, NMDA, 5HT_1A_, AMPA, GABA_A_ receptors. (6) Increased neuronal cells (Li et al., 2015). Neurons are the most basic structural and functional units in the nervous system. They are limited in number and difficult to recover once damaged. Acupuncture can increase the number of pyramidal neurons to improve synaptic function. (7) Improved vascular function (Gao et al., 2020). Vascular endothelial growth factor (VEGF) and angiogenin-1 (Ang-1) play an important role in angiogenesis. They can enhance vascular permeability through different receptors and maintain the integrity of the vascular lumen (Apte et al., 2019). Acupuncture can promote the repair of cerebral ischemia-reperfusion injury by upregulating VEGF and Ang-1. (8) Increased sugar metabolizing enzymes (Zhao et al., 2011). For brain cells, glucose metabolism is an important energy source. Glucose metabolism is closely related to enzymes (Zhang et al., 2021). Acupuncture increased the activities of hexokinase, pyruvate kinase, and glucose 6 phosphate dehydrogenase, thus affecting the energy metabolism system (Figure 4).

### Medicine can significantly improve cognition in VD rats, and there is no significant difference compared to acupuncture

The cholinesterase inhibitors such as donepezil, modulators of the NMDA receptor such as memantine, and calcium antagonists such as nimodipine were most commonly used for the treatment of VD currently (Sun, 2018). However, all the literatures included in this study using nimodipine tablets only, and no literature using donepezil for the treatment of VD meets the inclusion criteria. This ensures the uniformity of the drugs. This study showed that the efficacy of nimodipine is comparable to that of acupuncture. The mechanism of drug taking effect is also the same with that of acupuncture. This proves from another aspect that acupuncture is feasible as a therapy for VD.

The key factors of the drug include the dose, duration of use, and time to begin taking the drug (Battle et al., 2021). The dose of the drugs in the literature included in this study are mainly 12 mg/kg, but the treatment course of the medication has not been unified, mainly 14 days and 20 days. Therefore, we believed that the bias may be caused by the irregular medications. The correct time is closely associated with the recovery of the disease. Sometimes, taking the medicine too early may not have formed VD, and taking the medicine too late may miss the peak of disease development (Mijajlović et al., 2017). The literatures included in this study started the medication 7–15 days after modeling, and this imbalance may also be the cause of bias.

To promote the development of medical evidence for VD, researchers can conduct an appropriate animal study of donepezil vs. acupuncture in the treatment of VD, start drug treatment at an appropriate time, and take a full course of medication, providing an objective and diversified basis for clinicians to choose.

### The non-acupoints had little effect on the cognitive improvement of VD rats, which was similar to the model group

Non-acupoints are a common control method for acupuncture therapy, and its purpose is to highlight the specificity of acupuncture points. Usually, non-acupoints is selected at a position away from the spine and common acupoints, and the frequency and method of sham acupuncture should be consistent with the acupuncture group. This meta-analysis included 15 studies using sham acupuncture or non-acupoint studies. Additionally, based on this research analysis, the bilateral hypochondrium, 10 mm above the iliac crest was mainly selected as the acupuncture points in the control groups. (Ma et al., 2020) speculated that the effect of sham acupuncture is not good, mainly because (1) Due to their distance from the main nerve, stimulation signals from non-acupoints may not reach the central nervous system successfully (2) Signals are transmitted to the brain or spinal cord from non-acupoint acupuncture. However, they may not be effective acupoints for cognitive dysfunction (non-specific effects).

The results of this meta-analysis showed that the effect of non-acupoints was not statistically different from that of the impaired group and was inferior to the other groups. To highlight the specificity of the point of acupuncture, we believed that the bilateral hypochondrium, 10 mm above the iliac crest can be selected as the acupuncture points in the sham intervention group.

### Palliative therapy in the model group did not significantly improve the cognition of VD rats

The impaired group was treated with palliative therapy, that is, after surgical modeling, they maintained the same feeding and grasping as the rest of the interventions, but no drugs and acupuncture were given. The surgical modeling methods involved in this study include 2VO, 4VO, EO, and MCAO. The statistical results show that MCAO has the highest success rate, followed by 2VO and EO, and 4VO the lowest. All the methods can successfully shape VD animal models. MCAO is used mainly for modeling ischemic stroke, and the remaining three methods are used mainly for the establishment of chronic ischemia models. Therefore, we paid more focus on the differences of VD rats recovery capacity after successful modeling with different modeling methods. The recovery ability of VD rats is mainly associated to the operation method and the postoperative rest time. 4VO is a modeling method that has a greater effect on rat trauma and death, and the recovery difficulty of this method should be greater than that of 2VO, EO, and MCAO (Ganesana and Venton, 2021). Tuo et al. (2021) found that 8-week to 3-month resting after modeling, cerebral blood flow in rats could restore to normal. However, some studies suggested that the cognitive ability of rats was impaired at 4 weeks after 2VO modeling and the most serious damage was found at 20 weeks (Liu et al., 2005). Unfortunately, we investigated the specificity of acupuncture in this present study, while more in-depth discussions were conducted for the rest after modeling. This is also the next step for our team.

The results of this meta-analysis showed that the model group had the worst effect. We recommend choosing 2VO to create a VD rat model. After the modeling operation, postoperative rest and drug intervention time should be strictly mastered, which can better reflect the pathological characteristics of VD.

### Strengths and weaknesses

#### Advantage

This meta-analysis was performed based on the analysis under the Bayesian Markov Chain Monte Carlo framework. The Bayesian method refers to the posterior probability, which is more realistic and the estimated value is more accurate (Hu et al., 2020). This study is the first network meta-analysis of acupuncture treatment of VD animal models. We comprehensively analyzed various factors of acupuncture efficacy which included in the characteristics of the literature, and provide evidence-based medicine for future animal studies for acupuncture methods. The ultimate goal of all animal researches on acupuncture is to serve the clinic. According to the conclusion from this meta-analysis, the effect mechanism of head acupoints and some acupuncture points is comprehensively summarized. In this way, it may end the VD treatment predicament and improve the effect of clinical treatment, and bring new hope for VD patients.

#### Limitations

No study is perfect and this meta-analysis has inherent shortcomings. First, the difference between the syndrome differentiation and treatment followed by Chinese medicine in clinic and the generalized modeling method of animal research was an unavoidable drawback. Second, many literatures were not included in this analysis due to the strict literature inclusion criteria initially established. Third, the literatures included in this study were all carried out in China, resulting in an insufficient original literature for some interventions. Finally, the morris water maze was used as the outcome in this study, and some electron microscopy and light microscopy results were lacking. These shortcomings will partially bias the results of this analysis.

### Looking to the future

Evidence-based medicine research will never stop and good animal systematic review research should be linked to clinical practice. In the future, first of all, we will continue to conduct researches on diseases that conform to TCM syndromes combined with VD animal models, organically combine animal experiments with clinical experiments, and refer to each other to promote the transformation between preclinical evidence and clinical evidence. Second, the inclusion criteria will be reestablished and a comprehensive analysis will be performed on the amount of acupuncture stimulation, electron and light microscopy results, as well as the physical and chemical indicators. Finally, we hope that there will be more standardized, large-sample, high-quality animal studies in the future to verify the conclusions of this meta-analysis, provide more reliable reference for acupuncture clinicians, and efficiently reduce the physical and mental burden of VD patients.

## Conclusions

The results of this comprehensive network meta-analysis revealed that medication and acupuncture significantly altered outcomes of the Morris water maze in VD rats, while non-acupuncture and palliative care in the impaired group did not show efficacy. When conducting the experimental study for the treatment of VD rats with acupuncture, the acupuncture treatment plan of GV20 + ST36, MA, 14 days treatment can be used to obtain better results. In addition, this study provided multiple beneficial mechanisms about how acupuncture protects neurons in experimental VD, including antioxidative stress, reduction of neuroinflammation, inhibition of apoptosis, and increase in neurotransmitters, synaptic plasticity, and neuronal number. Our findings provide new information on animal studies of VD and contribute to the selection of protocols for acupuncture treatment of VD in animal experiments.

## Data availability statement

The original contributions presented in the study are included in the article/Supplementary materials, further inquiries can be directed to the corresponding author/s.

## Author contributions

WJ, JL, and ML designed the study and revised the manuscript for important intellectual content. YS, LZ, and TW acquired the data. CY and HL analyzed and interpreted the data. GL drafted the manuscript. All authors read and approved the final manuscript.

## Funding

This research was supported by two National Natural Science Foundation of China Youth Fund Projects (82104757 and 82004450).

## Conflict of interest

The authors declare that the research was conducted in the absence of any commercial or financial relationships that could be construed as a potential conflict of interest.

## Publisher's note

All claims expressed in this article are solely those of the authors and do not necessarily represent those of their affiliated organizations, or those of the publisher, the editors and the reviewers. Any product that may be evaluated in this article, or claim that may be made by its manufacturer, is not guaranteed or endorsed by the publisher.
