# Supplementary material for: Efficacy of acupuncture in animal models of vascular dementia: A systematic review and network meta-analysis
Source: Front Aging Neurosci. 2022 Aug 18;14:952181. doi: 10.3389/fnagi.2022.952181 (PMC9434110; doi:10.3389/fnagi.2022.952181)
Supplement: Supplementary file 1 [file Data_Sheet_1.docx]

**Supplementary Material Contents**

**Table 1: Study Characteristics··························································································2**

**Table 2: Protocols of acupuncture····················································································· 6**

**Table 3: Pair-wise meta-analysis of MWM···········································································8**

**Figure 1: Screening Process·····························································································9**

**Figure 2: Risk of bias graph·························································································· ··10**

**Figure 3: Risk of bias summary························································································11**

**Figure 4: Subgroup analysis of Escape latency·····································································12**

**Figure 5: Node split models·····························································································13**

**Figure 6: Rank probability of the time spent in the target quadrant··········································14**

**Search strategy············································································································15**

| **Table 1: Study Characteristics** | | | | | | | | | | | | | |
| --- | --- | --- | --- | --- | --- | --- | --- | --- | --- | --- | --- | --- | --- |
| **Study**  **(years)** | **Species**  **(sex)** | **Age** | **Weight**  **(g)** | **Model**  **method** | **Rest time (days)** | **Interventions**  **(animal numbers)** | | | | | | **Drug dose** | **MWM outcomes** |
| XU Pan  202204(Xu et al.,2022) | Wistar rats  (male) | 8  weeks | 280 ± 20 | 2VO | —— | Normal  (12) | S2VO  (12) | 2VO  (12) | MA  (12) | —— | —— | —— | (i)Escape latency  (ii)Number of crossings  (iii)Time spent in the target quadrant |
| CHEN Dan-Feng  202203(Chen et al.,2022) | SD rats  —— | —— | 220-250 | 2VO | 42 | —— | S2VO  (10) | 2VO  (10) | EA  (10) | —— | —— | —— | (i)Escape latency |
| BU Yu  202202(Bu et al.,2022) | SD rats  (male) | —— | 180-200 | 4VO | 7 | —— | S4VO  (6) | 4VO  (6) | EA  (6) | —— | —— | —— | (i)Escape latency  (ii)Number of crossings |
| Li-Longchun  202110(Li et al.,2021) | Wistar rats  (male) | 4-5  months | 300-320 | 2VO | 3 | —— | S2VO  (18) | 2VO  (18) | MA  (18) | —— | Non-  acupoint  (18) | —— | (i)Escape latency |
| Pan Pan  202107(Pan et al.,2021) | Wistar rats  (male) | 2  months | 260-280 | 2VO | 60 | Normal  (10) | S2VO  (10) | 2VO  (10) | MA  (10) | —— | Non-  acupoint  (10) | —— | (i)Escape latency  (ii)Number of crossings  (iii)Time spent in the target quadrant |
| Hui-Ling Wang  202106(H. L. Wang et al.,2021) | SD rats  (male) | 10-12  weeks | 260 ± 20 | MACO | 2  hours | —— | SMCAO  (12) | MCAO  (9) | EA  (9) | —— | —— | —— | (i)Escape latency  (ii)Number of crossings |
| Yan Cao  202102(Cao et al.,2021) | Wistar rats  (male) | 7-8  weeks | 260-300 | 2VO | 3 | —— | S2VO  (5) | 2VO  (5) | MA  (5) | —— | —— | —— | (i)Escape latency  (iii)Time spent in the target quadrant |
| Si-Ming Ma  202011(Ma et al.,2020) | Wistar rats  (male) | —— | 260-280 | 2VO | 3 | —— | S2VO  (8) | 2VO  (8) | MA  (8) | —— | NON- acupoint  8 | —— | (i)Escape latency  (ii)Number of crossings  (iii)Time spent in the target quadrant |
| GAO Yin-lai  202010(Gao et al.,2020) | SD rats  (male) | 6  weeks | 200-250 | 2VO | 14 | —— | S2VO  (6) | 2VO  (6) | MA  (6) | Nimodipine  (6) | —— | 0.062  g/kg | (i)Escape latency  (ii)Number of crossings |
| Na-Na Yang  202010(Yang et al.,2020) | Wistar rats  (male) | adult | 200-300 | 2VO | —— | —— | S2VO  (6) | 2VO  (6) | MA  (6) | —— | —— | —— | (i)Escape latency  (iii)Time spent in the target quadrant  (iv)Swimming speed |
| Yu zheng  202009(Zheng et al.,2020) | SD rats  (male) | —— | 270 ± 20 | MACO | 1 | —— | SMCAO  (10) | MCAO  (10) | EA  (10) | —— | —— | —— | (i)Escape latency  (iii)Time spent in the target quadrant |
| Lu Wang 202008(L. Wang et al.,2020) | Wistar rats  (male) | —— | 270-320 | 2VO | 3 | —— | S2VO  (6) | 2VO  (6) | MA  (6) | —— | —— | —— | (i)Escape latency  (iv)Swimming speed |
| Zhifu Wang  202003(Z. Wang et al.,2020) | SD rats  (male) | —— | 260–280 | MACO | 1 | —— | SMCAO  (18) | MCAO  (18) | EA  (18) | —— | NON- acupoint  18 | —— | (i)Escape latency  (ii)Number of crossings  (iv)Swimming speed |
| GUO Fei  202001(Guo et al.,2020) | SD rats  (male) | —— | 200±20 | 2VO | 3 | —— | S2VO  (10) | 2VO  (10) | EA  (10) | —— | —— | —— | (i)Escape latency  (ii)Number of crossings |
| YANG Xiao-bo  201910(Yang et al.,2019) | SD rats  (male) | adult | 200-230 | 2VO | 7 | Normal  (10) | —— | 2VO  (10) | MA  (10) | Nimodipine  (10) | —— | 1.0ML/100g | (i)Escape latency  (ii)Number of crossings |
| XUAN SU 201901(Su et al.,2019) | SD rats  (male) | 2  months | 220±20 | MACO | 10 |  | SMCAO  (10) | MCAO  (10) | MA  (10) | Nimodipine  (10) | —— | 20 mg/kg | (i)Escape latency  (ii)Number of crossings |
| Jing-Wen Yang  201812(Yang et al.,2018b) | Wistar rats  (male) | 8  weeks | 280-320 | 2VO | 3 | —— | S2VO  (6) | 2VO  (6) | MA  (6) | —— | Non-  Acupoint  (6) | —— | (i)Escape latency  (iv)Swimming speed |
| Wen Zhu  201805(Zhu et al.,2018) | Wistar rats  (male) | —— | 200–220 | 2VO | 3 | —— | S2VO  (10) | 2VO  (10) | MA  (10) | —— | Non-  acupoint  (10) | —— | (i)Escape latency  (iii)Time spent in the target quadrant |
| JIAN HE  201802(He et al.,2018) | SD rats  (male) | 2  months | 260±20 | MCAO | 7 | —— | SMCAO  (12) | MCAO  (12) | EA  (12) | —— | —— | —— | (i)Escape latency  (ii)Number of crossings |
| Si-Qi Du  201801(Du SQ et al.,2018) | Wistar rats  (male) | 10  weeks | —— | 2VO | 3 | —— | —— | 2VO  (10) | MA  (10) | —— | —— | —— | (i)Escape latency |
| Ruhui Lin  201709(Lin et al.,2017) | SD rats  (male) | 3  Months | 260-300 | MACO | 1 | —— | SMCAO  (10) | MCAO  (10) | EA  (10) | —— | —— | —— | (i)Escape latency  (ii)Number of crossings |
| Jiao Liu  201708(Liu et al.,2017) | SD rats  (male) | 10-12 weeks | 250-280 | MACO | 2 | —— | SMCAO  (10) | MCAO  (8) | EA  (9) | —— | —— | —— | (i)Escape latency  (ii)Number of crossings  (iv)Swimming speed |
| Zhang Zheng  201706(Zhang et al.,2017) | SD rats  (male) | —— | 180-220 | 4VO | 7 | —— | S4VO  (8) | 4VO  (8) | EA  (8) | —— | —— | —— | (i)Escape latency  (ii)Number of crossings |
| Yang Ye  201704(Ye & Li et al.,2017) | Wistar rats  (male) | —— | 270-300 | 2VO | 3 | —— | S2VO  (8) | 2VO  (8) | MA  (8) | —— | Non-  acupoint  (8) | —— | (i)Escape latency  (iii)Time spent in the target quadrant |
| Dexiong Han  201704(Han et al.,2017) | SD rats  (male) | 3-6  months | 200-220 | 2VO | —— | —— | S2VO  (10) | 2VO  (10) | EA  (10) | —— | —— | —— | (i)Escape latency  (iii)Time spent in the target quadrant |
| JIANG Ling-ge  201702(Jiang et al.,2017) | SD rats  (male) | adult | 200-240 | 4VO | 5 | —— | S4VO  (8) | 4VO  (8) | EA  (8) | —— | —— | —— | (i)Escape latency  (ii)Number of crossings |
| Hui Li  201601(Li et al.,2016) | Wistar rats  (male) | adult | 270-320 | 2VO | 3 | —— | S2VO  (8) | 2VO  (8) | MA  (8) | —— | Non-  acupoint  (8) | —— | (i)Escape latency  (iii)Time spent in the target quadrant |
| Xue-Rui Wang  201512(Wang et al.,2015) | Wistar rats  (male) | —— | 200-220 | 2VO | 3 | —— | S2VO  (10) | 2VO  (10) | MA  (10) | —— | Non-  acupoint  (10) | —— | (i)Escape latency  (iv)Swimming speed |
| Fang Li  201504(Li et al.,2015) | Wistar rats  (male) | adult | 320-360 | EO | 7 | Normal  (10) | SEO  (10) | EO  (11) | MA  (11) | —— | Non-  acupoint  (11) | —— | (i)Escape latency  (iv)Swimming speed |
| TIAN Wen-jing  201502(Tian et al.,2015) | SD rats  (male) | —— | 280±20 | 4VO | 7 | Normal  (10) | S4VO  (10) | 4VO  (8) | MA  (8) | —— | —— | —— | (i)Escape latency  (ii)Number of crossings |
| Junli Yang  201406(Yang et al.,2014) | Wistar rats  (male/  female) | —— | 200-250 | 2VO | 10 | —— | S2VO  (12) | 2VO  (12) | MA  (12) | —— | —— | —— | (i)Escape latency |
| Xuezhu Zhang  201401(Zhang et al.,2014) | Wistar rats  (male) | —— | 300–320 | EO | 7 | Normal  (10) | SEO  (10) | EO  (10) | MA  (10) | —— | Non- acupoint  10 | —— | (i)Escape latency  (ii)Number of crossings |
| Yanzhen Zhu  201307(Zhu et al.,2013) | SD rats  (female) | 12  months | 432±30 | 2VO | 30 | —— | S2VO  (6) | 2VO  (6) | EA  (6) | —— | —— | —— | (i)Escape latency |
| Xiaodong Feng  201305(Feng et al.,2013) | SD rats  (male) | —— | 250-280 | MACO | 2  hours | —— | SMCAO  (15) | MCAO  (15) | EA  (15) | —— | —— | —— | (i)Escape latency  (ii)Number of crossings |
| Yanzhen Zhu  201201(Zhu et al.,2012) | SD rats  (female) | 12  months | 400 ± 30 | 2VO | 30 | —— | S2VO  (8) | 2VO  (10) | EA  (12) | —— | —— | —— | (i)Escape latency |
| Dengming Wei  201110(Wei et al.,2011) | SD rats  (male/  female) | —— | 200-250 | 2VO | 7 | —— | S2VO  (10) | 2VO  (10) | EA  (10) | Nimodipine  (10) | —— | 12  mg/kg | (i)Escape latency  (ii)Number of crossings |
| Lan Zhao  201105(Zhao et al.,2011) | Wistar rats  (male) | 4  months | 240±20 | EO | 7 | Normal  (10) | SEO  (10) | EO  (10) | MA  (10) | —— | Non-  acupoint  (10) | —— | (i)Escape latency |
| Niu Wen-min  200904(Niu et al.,2009) | SD rats  (male) | adult | 300±20 | 4VO | —— | Normal  (10) | —— | 4VO  (10) | EA  (10) | —— | —— | —— | (i)Escape latency  (ii)Number of crossings |
| LIN Sui-jin  200810(Lin et al.,2008) | SD rats  (male) | 12  months | 620±80 | 2VO | 42 | Normal  (10) | S2VO  (10) | 2VO  (10) | EA  (10) | —— | Non-  acupoint  (10) | —— | (i)Escape latency  (iii)Time spent in the target quadrant |
| Ying Shao  200803(Shao et al.,2008) | SD rats  (male) | adult | 180-220 | 4VO | 10 | Normal  (8) | —— | 4VO  (8) | EA  (9) | Nimodipine  (8) | —— | 20  ml/kg | (i)Escape latency  (ii)Number of crossings |
| Wang Li  200702(Li et al.,2007) | SD rats  (male/  female) | 2-3  months | 200-250 | 4VO | 10 | —— | S4VO  (10) | 4VO  (11) | EA  (12) | —— | —— | —— | (i)Escape latency  (ii)Number of crossings |
| Wang Li  200406(Wang et al.,2004) | SD rats  (male/  female) | 2-3  months | 200-250 | 4VO | 10 | —— | S4VO  (10) | 4VO  (13) | EA  (14) | Nimodipine  (13) | —— | 12  mg/kg | (i)Escape latency  (ii)Number of crossings |

Abbreviations: SD rat (Sprague–Dawley rat); 2VO (bilateral common carotid artery occlusion); 4VO (4-vessel occlusion); MCAO (middle cerebral artery occlusion); EO (embolic occlusion); S2VO (Sham-2VO); S4VO (Sham-4VO); SMCAO (Sham-MCAO); SEO (Sham-EO); MA (Manual acupuncture); EA (Electroacupuncture); MWM (Morris Water Maze); Escape latency (Escape latency of each group in the hidden platform trial); Number of crossings (number of crossings over the former platform location); Time spent in the target quadrant; Swimming speed (swimming speed to reach the hidden platform in the hidden platform trial).

| **Table 2: Protocols of acupuncture** | | | | | |
| --- | --- | --- | --- | --- | --- |
| **Study(years)** | **Acupuncture** | **Acupoints** | **No-acupoints** | **Time** | **Course(days)** |
| Li-Longchun  202110 | MA | Baihui(GV20), bilateral Zusanli(ST36) | On the bilateral hypochondrium, 10 mm above iliac crest | —— | 14 |
| Yan Cao 202102 | MA | Baihui(GV20), bilateral Zusanli(ST36) | —— | 10min | 14 |
| Si-Ming Ma  202011 | MA | Baihui(GV20),bilateral Zusanli(ST36) | On the bilateral hypochondrium, 10 mm above iliac crest | 15min | 14 |
| Na-Na Yang202010 | MA | Baihui(GV20), bilateral Zusanli(ST36) | —— | 10min | 14 |
| Lu Wang 202008 | MA | Baihui(GV20), bilateral Zusanli(ST36) | —— | 10min | 14 |
| Jing-Wen Yang201812 | MA | Baihui(GV20), bilateral Zusanli(ST36） | On the bilateral hypochondrium, 10 mm above iliac crest | 30s | 14 |
| Wen Zhu 201805 | MA | Baihui (GV20), bilateral Zusanli (ST36) | 2 cm upper to the anterior superior spine | 10min | 14 |
| Si-Qi Du  201801 | MA | Baihui (GV20), bilateral Zusanli (ST36) | On the bilateral hypochondrium, 10 mm above iliac crest | 30S | 14 |
| Yang Ye  201704 | MA | Baihui(GV20), bilateral Zusanli(ST36) | On the bilateral hypochondrium, 10 mm above iliac crest | 30s | 14 |
| Hui Li  201601 | MA | Baihui(GV20), bilateral Zusanli(ST36) | On the bilateral hypochondrium, 10 mm above iliac crest | 30s | 14 |
| Xue-Rui Wang 201512 | MA | Baihui(GV20), bilateral Zusanli(ST36) | 2 cm upper to the anterior superior spine | 1min | 14 |
| Zhang Zheng 201706 | EA | Baihui(GV20), Dazhui(GV14) | —— | 20min | 24 |
| Dexiong Han 201704 | EA | Baihui(GV20), Dazhui(GV14) | —— | 30min | 28 |
| JIANG Ling-ge201702 | EA | Baihui(GV20), Dazhui(GV14) | —— | 30min | 10 |
| Dengming Wei 201110 | EA | Baihui(GV20), Dazhui(GV14) | —— | 20min | 10 |
| Wang Li 200702 | EA | Baihui(GV20), Dazhui(GV14) | —— | 20min | 15 |
| Wang Li 200406 | EA | Baihui(GV20), Dazhui(GV14) | —— | 20min | 15 |
| Hui-Ling Wang 202106 | EA | Baihui(GV20), Shenting (GV24) | —— | 30min | 8 |
| Zhifu Wang 202003 | EA | Baihui (GV20), Shenting (GV24) | 2 cm upper to the anterior superior spine | 30min | 14 |
| JIAN HE 201802 | EA | Baihui (GV20), Shenting (GV24) | —— | 30min | 7 |
| Ruhui Lin 201709 | EA | Baihui (GV20), Shenting (GV24) | —— | 30min | 7 |
| Jiao Liu 201708 | EA | Baihui (GV20), Shenting (GV24) | —— | 30min | 7 |
| XiaodongFeng 201305 | EA | Baihui (GV20), Shenting (GV24) | —— | 30min | 10 |
| CHEN Dan-Feng  202203 | EA | Baihui (GV20), Dazhui (GV14), bilateral Shenshu (BL23) | —— | 30min | 28 |
| Yanzhen Zhu  201307 | EA | Baihui(GV20), Dazhui(GV14), bilateral Shenshu(BL23) | —— | 20min | 30 |
| Yanzhen Zhu  201201 | EA | Baihui (GV20), Dazhui (GV14), bilateral Shenshu (BL23) | —— | 20min | 30 |
| LIN Sui-jin  200810 | EA | Baihui(GV20), Dazhui(GV14), bilateral Shenshu(BL23) | at the thoraco-abdominal junction of the first and second lumbar vertebrae | 20min | 30 |
| XU Pan  202204 | MA | Danzhong (CV17), Qihai (CV6), Zhongwan (CV12), bilateral Zusanli (ST36), bilateral Xuehai (SP10) | —— | 30S | 15 |
| Pan Pan  202107 | MA | Danzhong (CV17), Qihai (CV6), Zhongwan (CV12), bilateral Zusanli (ST36), bilateral Xuehai (SP10) | On the bilateral hypochondrium, 3 mm above iliac crest | 30S | 14 |
| Xuezhu Zhang  201401 | MA | Danzhong (CV17), Qihai (CV6), Zhongwan (CV12), bilateral Zusanli (ST36), bilateral Xuehai (SP10) | On the hypochondrium, 3 mm above iliac crest. | 30S | 21 |
| Lan Zhao  201105 | MA | Danzhong (CV17), Qihai (CV6), Zhongwan (CV12), bilateral Zusanli (ST36), bilateral Xuehai (SP10) | On the bilateral hypochondrium, 10 mm above iliac crest | 30S | 21 |
| BU Yu  202202 | EA | Baihui (DV20), Danzhong (CV17), Geshu (BL17), Qihai (CV6) and Sanyinjiao (SP6) | located around the 5 acupoints, not located on the meridian route, and did not belong to the traditional 14 meridians | 30 min | 21 |
| GAO Yin-lai  202010 | MA | Mingmen (GV4), Dazhui (GV14), Fengfu (GV16), Baihui (GV20), Shenting (DU24), Shuigou (GV26), bilateral Neiguan (PC6), bilateral Daling (PC7), bilateral Laogong (PC8) | —— | 30min | 14 |
| Yu Zheng202009 | EA | Baihui (GV20), Yingtang (EX-HN3) |  | 10min | 14 |
| GUO Fei  202001 | EA | Baihui (GV20), Dazhui (GV14), Geshu (BL17), Housanli | —— | 10min | 14 |
| YANG Xiao-bo201910 | MA | Baihui (GV20), Dazhui (GV14), Shuigou (GV26) | —— | 30min | 14 |
| XUAN SU  201901 | MA | Baihi (GV20), Dazhui (GV14), Shuigou (GV26), Fengfu (GV16) |  | 20min | 15 |
| Fang Li  201504 | MA | bilateral Zusanli (ST36) | On the bilateral hypochondrium, 10 mm above iliac crest | 30s | 14 |
| TIAN Wen-jing201502 | MA | Daomoshangjiao,Jiyi,Siwei | —— | 30min | 10 |
| Junli Yang 201406 | MA | Cluster-Needling | —— | 6hours | 28 |
| Niu Wen-min200904 | EA | Yingtang (EX-HN3), Yingxiang (LI20) | —— | 10min | 49 |
| Ying Shao  200803 | EA | Baihui (GV20), Dazhui (GV14), bilateral Geshu (BL17), bilateral Pishu (BL20) | —— | 20min | 15 |

Abbreviations: MA (Manual acupuncture); EA (Electroacupuncture)

| **Table 3: Pair-wise meta-analysis of MWM** | | | | | |
| --- | --- | --- | --- | --- | --- |
| **Escape Latency** | | | | | |
| Intervention | Ga VS Gi | Ga VS Gna | Ga VS Gn | Ga VS Gs | Ga VS Gm |
| MD  （95% Crl） | 25.73  (22.23, 29.24) | 14.71  (6.35, 23.07) | -13.65  (-20.23, -7.08) | -7.72  (-9.86, -5.58) | 2.40  (-5.04, 9.85) |
| Intervention | Gi VS Gm | Gi VS Gn | Gi VS Gs | Gi VS Gna | Gna VS Gs |
| MD  （95% Crl） | -41.60  (-51.06, -32.14) | -41.26  (-55.55, -26.97) | -30.68  (-34.22, -27.15) | -3.31  (-9.35, 2.74) | -20.23  (-24.86, -15.59) |
| Intervention | Gm VS Gs | Gm VS Gn | Gn VS Gna | Gn VS Gs |  |
| MD  （95% Crl） | -9.83  (-12.72, -6.94) | -11.34  (-28.38, 5.70) | 20.57  (11.83, 29.30) | 1.65  (0.44, 2.85) |  |
| **Number of crossings** | | | | | |
| Intervention | Ga VS Gi | Ga VS Gn | Ga VS Gs | Ga VS Gm | Ga VS Gna |
| MD  （95% Crl） | -2.65  (-3.29, -2.00) | 1.36  (0.60, 2.11) | 1.31  (0.76, 1.85) | -0.68  (-1.85, 0.50) | -0.71  (-1.26, -0.17) |
| Intervention | Gi VS Gm | Gi VS Gn | Gi VS Gs | Gi VS Gna | Gna VS Gs |
| MD  （95% Crl） | 2.99  (2.16, 3.82) | 3.55  (1.95, 5.14) | 3.83  (2.88, 4.77) | 0.31  (-0.35, 0.97) | 1.82  (0.65, 2.99) |
| Intervention | Gm VS Gs | Gm VS Gn | Gn VS Gna | Gn VS Gs |  |
| MD  （95% Crl） | 1.46  (1.00, 1.92) | 1.82  (0.07, 3.57) | -1.50  (-1.93, -1.06) | -0.08  (-0.43, 0.27) |  |
| **Time spent in the target quadrant** | | | | | |
| Intervention | Ga VS Gi | Ga VS Gna | Ga VS Gs | Ga VS Gn | Gna VS Gs |
| MD  （95% Crl） | -7.69  (-9.47, -5.92) | -7.06  (-10.10, -4.02) | 5.04  (3.70,6.39) | 8.23  (0.00,16.45) | 13.49  (8.38, 18.61) |
| Intervention | Gi VS Gs | Gi VS Gn | Gi VS Gna | Gn VS Gna | Gn VS Gs |
| MD  （95% Crl） | 13.25  (10.16, 16.34) | 17.57  (1.90, 33.24) | 1.34  (-0.00, 2.68) | -21.14  (-48.77, 6.49) | -2.04  (-6.88, 2.80) |
| **Swimming speed** | | | | | |
| Intervention | Ga VS Gi | Ga VS Gn | Ga VS Gna | Ga VS Gs | Gna VS Gs |
| MD  （95% Crl） | -0.32  (-1.48, 0.83) | -1.69  (-5.01, 1.63) | -0.13  (-1.35, 1.09) | 0.06  (-1.20, 1.32) | -0.03  (-1.51, 1.44) |
| Intervention | Gi VS Gn | Gi VS Gna | Gi VS Gs | Gn VS Gna | Gn VS Gs |
| MD  （95% Crl） | -1.85  (-4.88, 1.18) | 0.19  (-1.00, 1.37) | 0.26  (-0.94, 1.47) | 3.38  (0.48,6.28) | 0.81  (-1.43, 3.05) |

Treatments, Efficacy (MD [95% Crl]), P<0.05; Efficacy (MD [95% Crl]), P>0.05;

Abbreviations: MWM (Morris Water Maze); Escape latency (Escape latency of each group in the hidden platform trial); Number of crossings (number of crossings over the former platform location); Time spent in the target quadrant; Swimming speed (swimming speed to reach the hidden platform in the hidden platform trial). Gn (Normal group), Gs (Sham-operated group), Gi (impaired group), Ga (acupuncture group), Gna (Non-acupoint group), Gm (Medicine group).

**Figure 1. Screening Process** (Overview of study screening and selection process according to PRISMA guidelines).


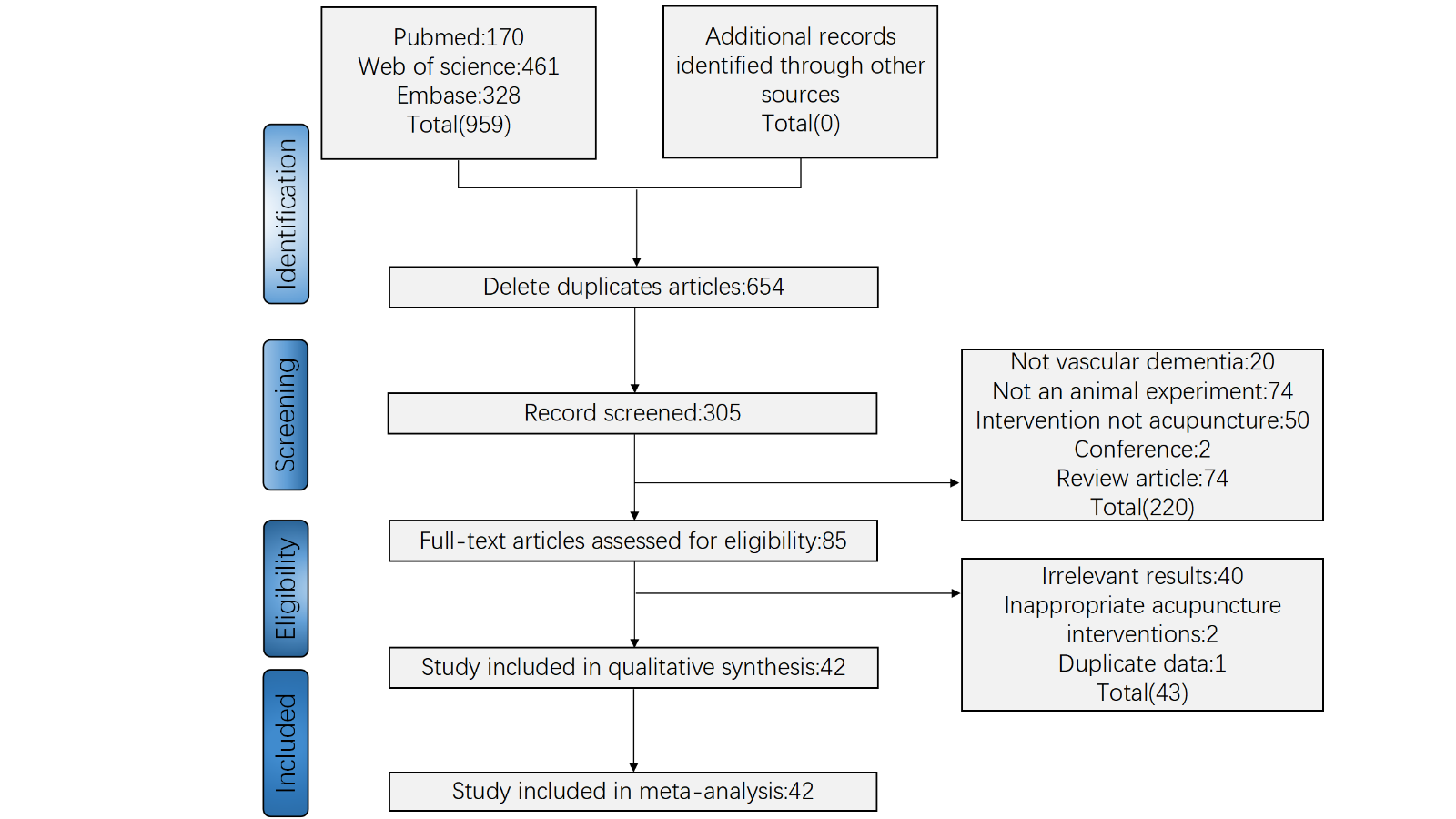


**Figure 2: Risk of bias graph**

**Figure 3: Risk of bias graph summary**

**Figure 4: Subgroup analysis of Escape latency**


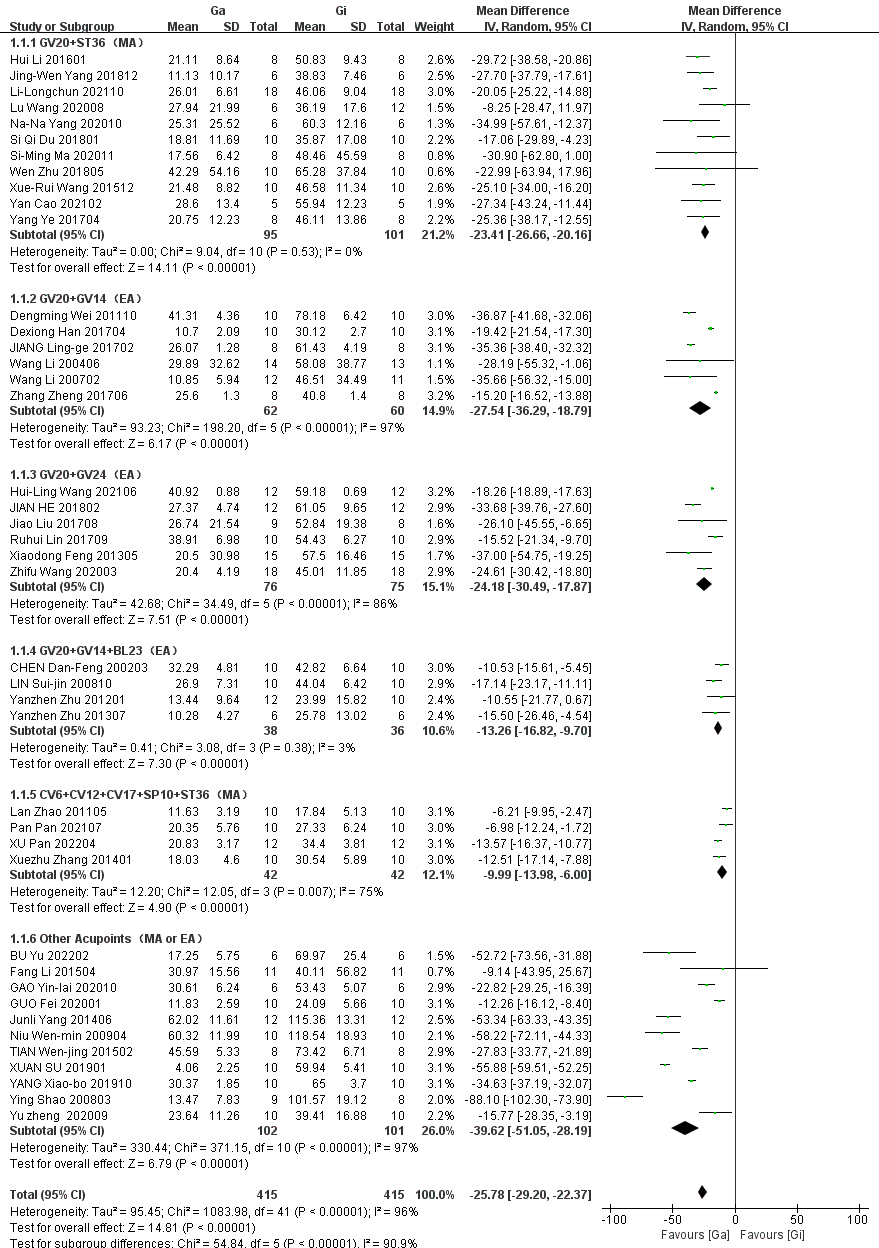


Abbreviations: Gi (Impaired group); Ga (acupuncture group); MA (Manual Acupuncture); EA (Electroacupucture); GV20 + ST36 (Baihui + bilateralZusanli); GV20 + GV14 (Baihui + Dazhui); GV20 + GV24 (Baihui + shenting); GV20 + GV14 + BL23 (Baihui + Dazhui + bilateral Shenshu); CV6 + CV12 + CV17 + SP10 + ST36 (Qihai + Zhongwan + Danzhong + bilateral Xuehai + bilateral Zusanli).

**Figure 5:Node split models of Escape latency and Number of crossings**

**(A) Escape latency**


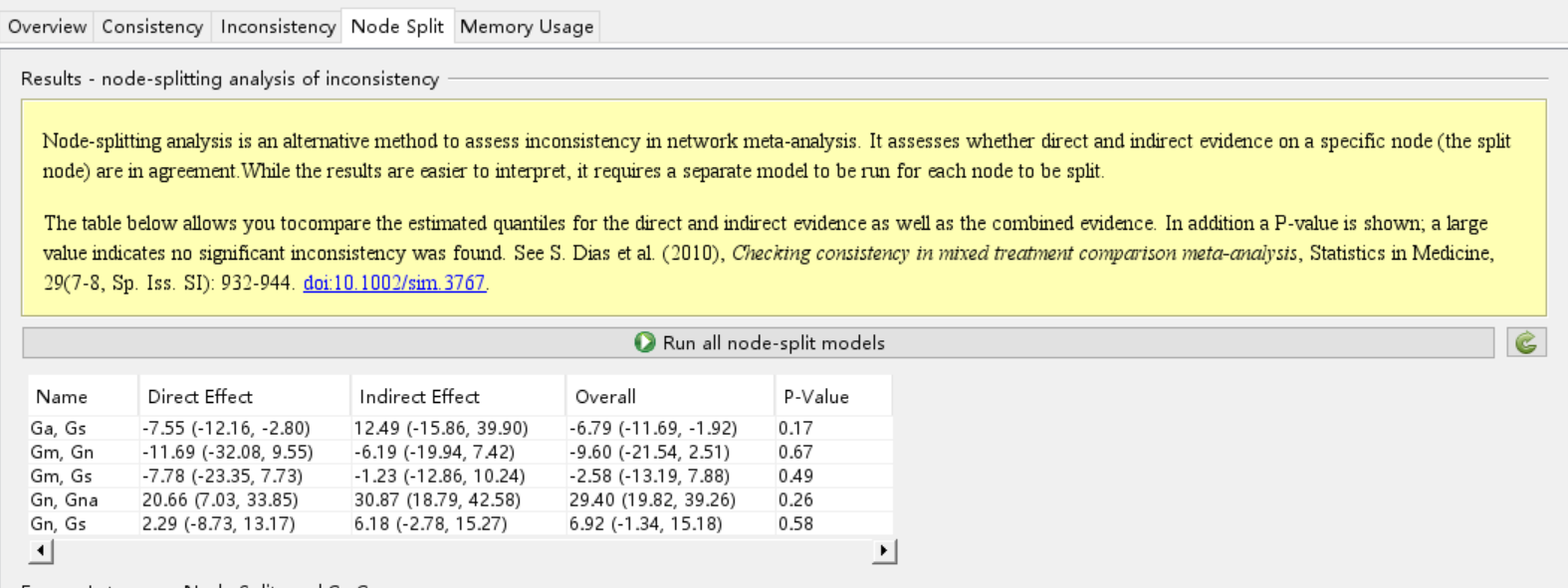


**(B) Number of crossings**


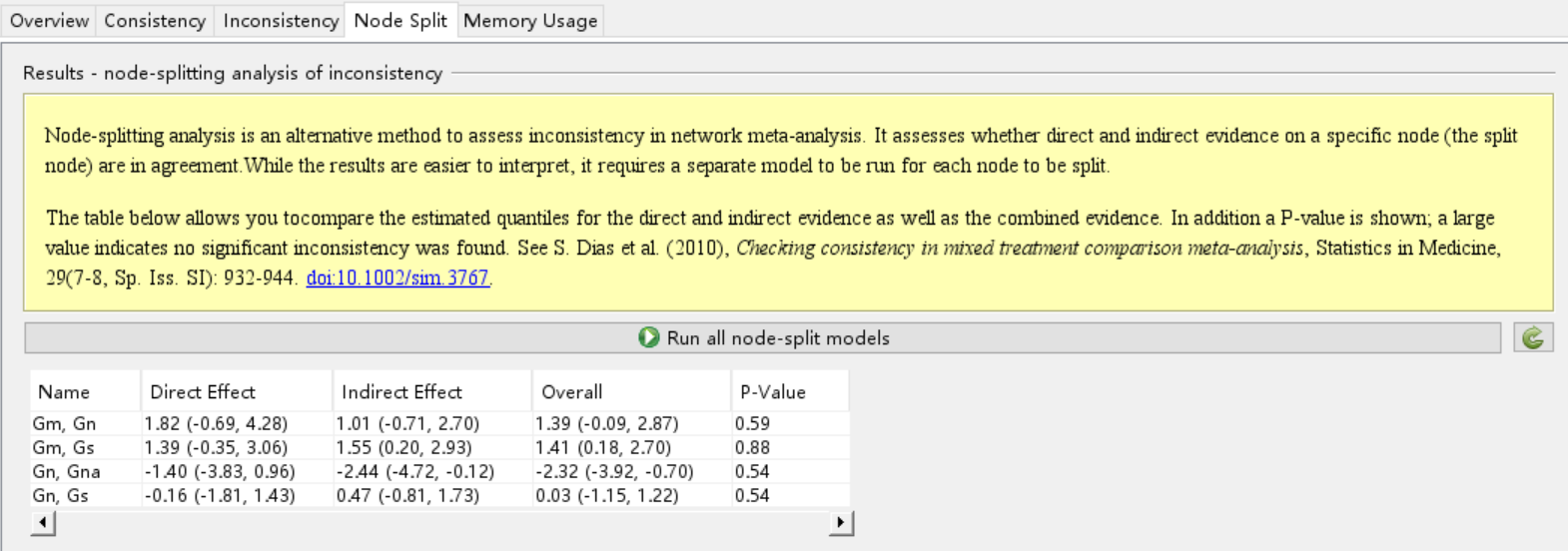


**(C)Time spent in target quadrant**


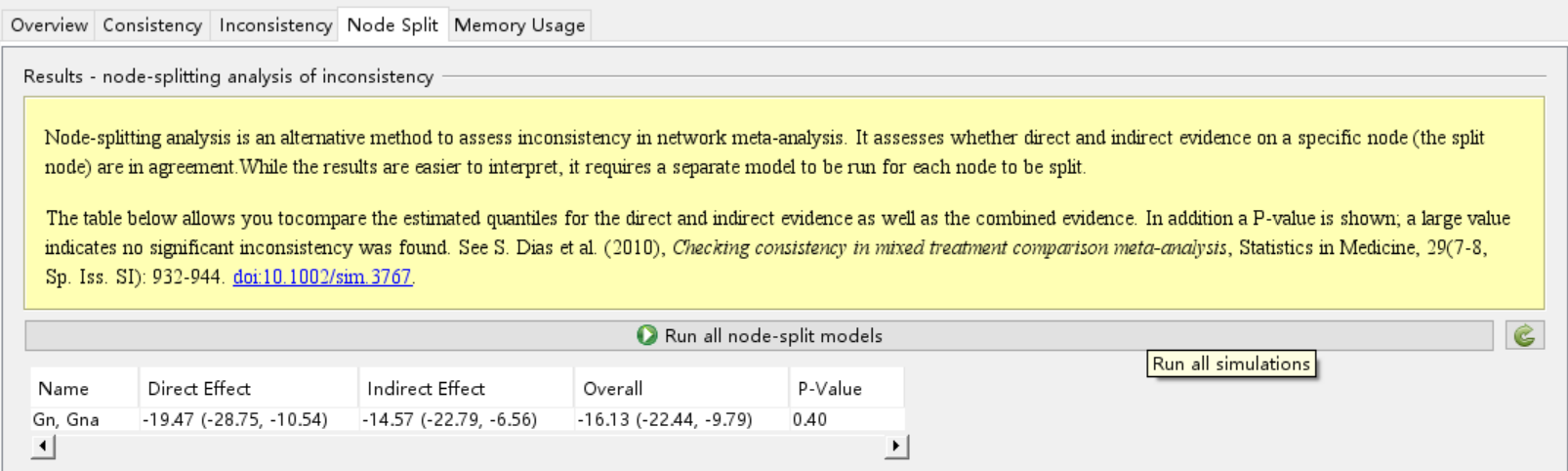


Abbreviations: Gn (Normal group), Gs (Sham-operated group), Gi (impaired group), Ga (acupuncture group), Gna (Non-acupoint group), Gm (Medicine group).

**Figure 6:Rank probability of the time spent in the target quadrant**


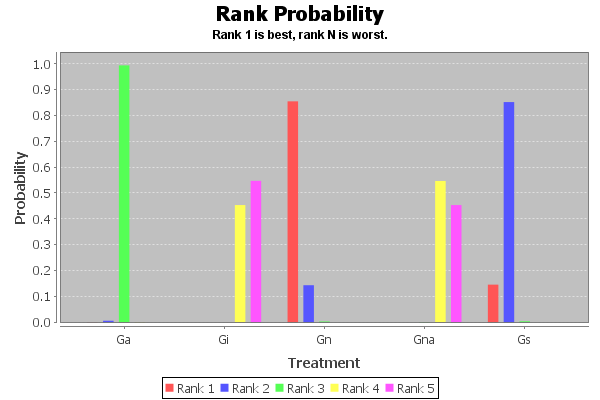


Abbreviations: Gn (Normal group), Gs (Sham-operated group), Gi (impaired group), Ga (acupuncture group), Gna (Non-acupoint group), Gm (Medicine group).

**Search strategy**

The search time is limited to the establishment of the database until April 2022. The search terms are: acupuncture, electroacupuncture, acupoint, vascular dementia, infarct dementia, vascular cognitive impairment. each search Word are used alone or in combination.

**Pubmed**

1. acupuncture
2. electroacupuncture
3. acupoint
4. vascular dementia
5. infract dementia
6. Vascular cognitive impairment

(acupuncture[Title/Abstract]) AND (vascular dementia[Title/Abstract])

(electroacupuncture[Title/Abstract]) AND (vascular dementia[Title/Abstract])

(acupoint[Title/Abstract]) AND (vascular dementia[Title/Abstract])

(acupuncture[Title/Abstract]) AND (infract dementia[Title/Abstract])

(electroacupuncture[Title/Abstract]) AND (infract dementia[Title/Abstract])

(acupoint[Title/Abstract]) AND (infract dementia[Title/Abstract])

(acupuncture[Title/Abstract]) AND (vascular cognitive impairment [Title/Abstract]) (electroacupuncture[Title/Abstract]) AND (vascular cognitive impairment [Title/Abstract]) (acupoint[Title/Abstract]) AND (vascular cognitive impairment [Title/Abstract])

**Embase**

1. acupuncture
2. electroacupuncture
3. acupoint
4. vascular dementia
5. infract dementia
6. Vascular cognitive impairment

acupuncture:ab,ti AND 'vascular dementia':ab,ti

electroacupuncture:ab,ti AND 'vascular dementia':ab,ti

acupoint:ab,ti AND 'vascular dementia':ab,ti

acupuncture:ab,ti AND ' infract dementia':ab,ti

electroacupuncture:ab,ti AND ' infract dementia':ab,ti

acupoint:ab,ti AND ' infract dementia':ab,ti

acupuncture:ab,ti AND 'vascular cognitive impairment ':ab,ti

electroacupuncture:ab,ti AND 'vascular cognitive impairment ':ab,ti

acupoint:ab,ti AND 'vascular cognitive impairment ':ab,ti

**Web of science (including Medline)**

1. acupuncture
2. electroacupuncture
3. acupoint
4. vascular dementia
5. infract dementia
6. Vascular cognitive impairment

(TS=(acupuncture)) AND TS=(vascular dementia)

(TS=(electroacupuncture)) AND TS=(vascular dementia)

(TS=( acupoint)) AND TS=(vascular dementia)

(TS=(acupuncture)) AND TS=(infract dementia)

(TS=(electroacupuncture)) AND TS=(infract dementia)

(TS=( acupoint)) AND TS=(infract dementia)

(TS=(acupuncture)) AND TS=(vascular cognitive impairment)

(TS=(electroacupuncture)) AND TS=(vascular cognitive impairment)

(TS=( acupoint)) AND TS=(vascular cognitive impairment)
